# Distribution, Prevalence, and Causative Agents of Fungal Keratitis: A Systematic Review and Meta-Analysis (1990 to 2020)

**DOI:** 10.3389/fcimb.2021.698780

**Published:** 2021-08-26

**Authors:** Kazem Ahmadikia, Sanaz Aghaei Gharehbolagh, Bahareh Fallah, Mahsa Naeimi Eshkaleti, Pooneh Malekifar, Saeedeh Rahsepar, Muhammad I. Getso, Savitri Sharma, Shahram Mahmoudi

**Affiliations:** ^1^Department of Medical Parasitology and Mycology, School of Public Health, Tehran University of Medical Sciences, Tehran, Iran; ^2^Department of Mycology, Faculty of Medical Sciences, Tarbiat Modares University, Tehran, Iran; ^3^Students’ Scientific Research Center, Tehran University of Medical Sciences, Tehran, Iran; ^4^Department of Epidemiology and Biostatistics, School of Public Health, Tehran University of Medical Sciences, Tehran, Iran; ^5^Department of Medical Microbiology and Parasitology, Faculty of Clinical Sciences, College of Health Sciences, Bayero University Kano, Kano, Nigeria; ^6^Jhaveri Microbiology Centre, L. V. Prasad Eye Institute, Hyderabad, India; ^7^Department of Parasitology and Mycology, School of Medicine, Iran University of Medical Sciences, Tehran, Iran

**Keywords:** *Aspergillus*, corneal ulcer, fungal keratitis, *Fusarium*, keratomycosis, meta-analysis, yeasts

## Abstract

**Objectives:**

This study aims to provide an overview of the prevalence, distribution, and causative agents of fungal keratitis.

**Methods:**

All the articles with data on the prevalence of fungal keratitis among various patient groups from January 1, 1990 to May 27, 2020 were retrieved through a systematic search in PubMed, Scopus, Web of Science, and Google Scholar. Data were extracted, and the pooled estimated prevalence of fungal keratitis, yeast/mold infection, the spectrum and frequency of various causative agents, and the pooled estimated prevalence of mixed infections were calculated in general and in various countries (wherever possible) using meta-analysis.

**Results:**

From 11,235 articles retrieved in the primary search step, 169 met the inclusion criteria. The 169 eligible articles were divided into six groups and analyzed separately. The pooled prevalence of fungal keratitis was variable with values ranging from 0.05% among postkeratoplasty patients to 43.01% among patients with a clinical suspicion of fungal keratitis. There was also a country-dependent variation in the prevalence (Paraguay: 50.1% (95% CI, 35.11, 65.00); Ireland: 1.1% (95% CI, 0.03, 6.04)). Except for postkeratoplasty cases (yeast: 51.80%), in all patient groups, molds were more common than yeasts. Although more than 50 distinct species of fungi have been found to cause fungal keratitis, *Fusarium* species followed by *Aspergillus* species were the most common causes of the disease. In general, 9.29% (95% CI, 6.52, 12.38) of fungal keratitis cases were mixed with bacterial agents.

**Conclusion:**

The prevalence of fungal keratitis can vary dramatically depending on the patient groups and geographical origin; however, the dominant causative agents are generally similar.

## Introduction

Keratitis describes a group of acute or chronic inflammatory disorders occurring in the cornea following any factors disrupting the protective mechanism of the outer layer of the eye (Acharya et al., 2017). The inflammation may be of allergic (reactive), physical, chemical, or infective (bacteria, fungi, parasites, and viruses) origin (Acharya et al., 2017). Infectious or microbial keratitis, one of the most serious eye diseases, has long been acknowledged as a leading cause of visual impairment and preventable blindness worldwide (Ghosh et al., 2016). Very appropriately, infectious keratitis is now included among neglected tropical diseases by the World Health Organization (WHO) (Ung et al., 2019). Due to the nonspecific signs and symptoms, and rapid progression, microbial keratitis is diagnostically challenging for physicians (Słowik et al., 2015).

The prevalence rate and epidemiological distribution of fungal keratitis (FK) are strongly associated with geographical locations and widely vary throughout the world, even between different regions of the same country and in different groups of individuals (Kredics et al., 2015). FK is globally getting increasing attention particularly in developing countries and tropical and subtropical regions (Srinivasan, 2004; Maharana et al., 2016) where approximately half of the world’s fungal keratitis cases occur (Thomas and Kaliamurthy, 2013); therefore, the contribution of FK, as one of the major causes of visual loss cannot be neglected (Garg et al., 2016).

Characteristic clinical features of FK have been described; however, they are not pathognomonic enough and often mimic bacterial or parasitic keratitis (Ghosh et al., 2016). Therefore, in absence of laboratory diagnosis, majority of cases may be treated empirically (Dahlgren et al., 2007; Ghosh et al., 2016), resulting in poor outcomes that may progress to endophthalmitis, particularly if left untreated (Thomas, 2003; Kredics et al., 2015; Bourcier et al., 2017). Emerging fungal pathogens and resistance to existing antifungal agents have further contributed to poor prognosis in FK (Kredics et al., 2015; Maharana et al., 2016).

A healthy cornea is rarely infected with fungal agents (Khater et al., 2014). Traditionally, FK is considered to be a disease prevalent in rural settings occurring in traumatized eyes by vegetative materials or soil-contaminated objects in middle-aged agriculturists and laborers. These are evidenced to be the most susceptible eyes for fungal infection of the cornea in low-income countries (Tuli, 2011; Acharya et al., 2017). Conversely, contact lens usage (CLU) is the primary culprit, predisposing hosts to FK in developed countries (Srinivasan, 2004; Tuli, 2011; Acharya et al., 2017).

The most prevailing species implicated in FK include those belonging to *Fusarium*, *Aspergillus*, *Candida*, *Curvularia*, and *Penicillium* genera in descending frequency (Gower et al., 2010; Revankar and Sutton, 2010; Chang and Chodosh, 2011; Ansari et al., 2013; Qiu et al., 2015). Most of these species are environmental residents that invade traumatized or immunologically weakened eyes (Maharana et al., 2016). However, thermally dimorphic fungi, although rarely, have also been reported as causative agents of FK in both healthy and immunocompromised eyes (Morrison et al., 2013; Thomas and Kaliamurthy, 2013). Yeast-associated FK occur more frequently in temperate climates whereas filamentous fungal etiology is primarily documented in regions with tropical weather (Mahmoudi et al., 2018).

In this context, there are several epidemiological studies regarding the frequency of fungal keratitis, their related risk factors, and the spectrum of etiological agents. In the era of the increasing number of immunocompromised populations and CLU, the prevalence of FK differs from country to country and even between regions of the same country. Nevertheless, there is a lack of a comprehensive study to define the most frequent causative agents of FK and to compare the prevalence rates of FK in different population-based studies in different countries. Therefore, we aimed to systematically review data pertaining to studies concerning FK in the English language between 1990 and 2020 to provide contemporary insights into the epidemiology and causative agents of FK.

## Methods

### Database Searching

The protocol of this study was registered in the International Prospective Register of Systematic Reviews (PROSPERO) with the ID number CRD42020188770, and the study was done according to the preferred reporting items for systematic reviews and meta-analyses (**Supplementary File 1**) (Moher et al., 2009). Relevant literatures were searched in Web of Science (ISI), PubMed, Scopus, and Google scholar using the main keywords “fungal keratitis,” “keratomycosis,” and “mycotic keratitis” and a set of other keywords, solely, and in combination (**Supplementary File 2**). To ensure that the search captures all the relevant articles and because of usage of general phrases such as “infectious keratitis” or “microbial keratitis” that include a set of microorganisms, i.e., bacteria, viruses, fungi, and amoebae, a wide search strategy was used. The search was limited to “article” as document type (whenever available), “English” as language, and “January 1, 1990 to the date of search (May 27, 2020)” as the publication date.

### Study Selection and Quality Assessment

The resulting articles from the searched databases were imported into the EndNote X9 software library for de-duplicating, and title and abstract screening. After excluding irrelevant citations, full texts of citations were downloaded and checked for eligibility. All studies reporting data of prevalence and causative agents of fungal keratitis were eligible. Studies reporting animal or *ex vivo* models of keratitis; case reports and case series (without a denominator of the population from which the cases had been diagnosed); case-controls; cohort studies; clinical trials; *in vitro* studies on virulence factors, antifungal susceptibility pattern of keratitis-isolated fungi but without data of prevalence; review articles; letters; and studies on therapy or keratitis caused by nonfungal microorganisms were excluded. Studies on specific populations, e.g., specific age groups, those with specific surgical interventions, etc. were also excluded except in cases where at least five articles on the same patient group were available. In this case, the articles were included in the study but were analyzed separately. The quality of the relevant full texts was assessed using a modified version of the Newcastle-Ottawa Scale (Herzog et al., 2013). All steps of screening and quality assessment were done by two independent researchers and in the case of inconsistency, a third researcher made the final decision.

### Data Extraction

The name of the first author and the year of publication along with the data of interest comprising country, continent, the total number of studied patients, the number of fungal keratitis cases, frequency of yeast and mold pathogens, frequency of various fungal genera and species (if they were identified), frequency of mixed fungal and bacterial infections, gender, and underlying conditions of confirmed patients (if available) were extracted by two independent researchers into a Microsoft Office Excel 2019 file.

### Statistical Analysis

Data were analyzed using Stata software version 14. To determine the heterogeneity, *I* ^2^ and Cochran *Q* test were used. In accordance with the Higgins classification approach, *I* ^2^ values above 0.7 were considered high heterogeneity. In the presence of heterogeneity, a random-effect model was used in calculations. The pooled prevalence with a 95% confidence interval (CI) was calculated using the “metaprop” command, and to estimate the pooled prevalence, we used the random-effect model. The exact method was used for calculating pooled estimates, variances, and their confidence intervals. We used Freeman-Tukey double arcsine transformation for variance stabilization.

The pooled prevalence of fungal keratitis and the pooled prevalence of yeast and mold keratitis and mixed fungal-bacterial infections were estimated. To determine the pooled prevalence of fungal keratitis in different countries, subgroup analysis was performed. The “metabias” command was used to check the publication bias, and if there was any publication bias, the prevalence rate was adjusted with the “metatrim” command using trim-and-fill method. The meta-regression analysis was used to examine the effect of the year of publication and sample size as factors affecting heterogeneity among studies. In all analyses, a significance level of 0.05 was considered.

## Results

**Figure 1:**
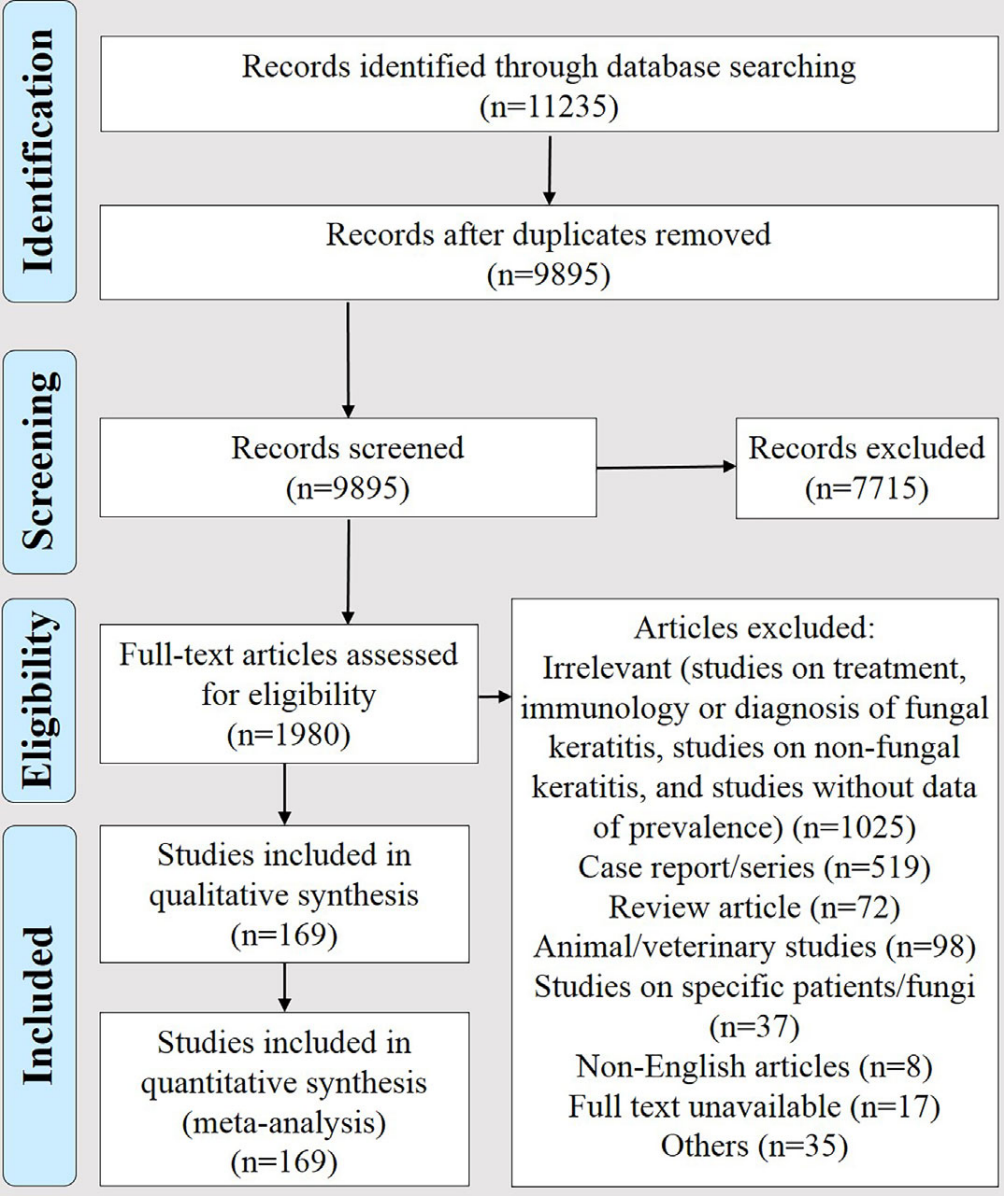
The PRISMA flow diagram of selecting studies reporting data on the prevalence of fungal keratitis between January 1, 1990 and May 27, 2020.

As presented in **Figure 1**, from 11,235 articles retrieved in the primary search step, 169 met the inclusion criteria (**Supplementary File 3**). Results of their quality assessment are presented in **Supplementary File 4**. These articles were divided into six groups, i.e., studies reporting data of fungal keratitis among (I) patients suspected of microbial keratitis (*n* = 109), (II) suspected of fungal keratitis (*n* = 13), (III) those with culture-confirmed microbial keratitis (*n* = 10), (IV) contact lens wearers (*n* = 6), (V) pediatric patients (*n* = 8), and (VI) those who underwent keratoplasty (*n* = 23) and analyzed separately. This practice was used for minimizing bias because the denominator was not identical in these groups. For instance, in group III, the denominator was very much smaller than other groups because it did not include patients who were clinically suspected and their etiology was noninfectious. The summary of pooled prevalence and the most common causative agents in each group is presented in **Table 1**.

**Table 1:** Summary of overall estimated prevalence values and the three most common causes of fungal keratitis among various groups of patients included in the present meta-analysis (1990 to 2020).

Patient group	*N* of studies	Overall estimated prevalence (95% confidence interval)	The most common etiologies		
			Fist rank	Second rank	Third rank
Clinically suspected microbial keratitis	109	23.64 (20.39–27.05)	*Fusarium* spp.	*Aspergillus* spp.	*Curvularia* spp.
Culture-confirmed microbial keratitis	10	17.89 (6.96–32.42)	*Fusarium* spp.	*Aspergillus* spp.	*Candida* spp.
Clinically suspected fungal keratitis	13	43.01 (30.88–55.59)	*Aspergillus* spp.	*Fusarium* spp.	*Curvularia* spp.
Pediatric patients (≤16 years)	8	14.88 (6.87–25.11)	*Fusarium* spp.	*Aspergillus* spp.	*Candida* spp.
Contact lens wearers	6	18.05 (1.04, 46.91)	*Fusarium* spp.	*Aspergillus* spp.	*Acremonium* spp.
Postkeratoplasty	16^a^	8.57 (3.89–14.62)	*Fusarium* spp.	*Aspergillus* spp.	*Candida* spp.
	7^b^	0.05 (0.00–0.14)			

a Keratoplasty due to infective keratitis.

b Keratoplasty due to a variety of indications except infective keratitis.

Regarding the origin of studies, from the 169 articles, 124 (72.94%) have been reported from Asia, 24 (14.12%) from America, nine (5.29%) from Africa, six (5.29%) from Europe, and four (2.35%) from Oceania. In one article, two sets of data, one from India and one from Ghana have been reported; accordingly, they were treated as different studies in the calculation of origin of studies. Regarding the country of studies, the majority of studies have been reported from India (*n* = 56, 32.94%), followed by China (*n* = 16, 9.41%), USA (*n* = 15, 8.82%), and Nepal (*n* = 13, 7.65%). The distribution and frequency of studies from various countries are shown in **Table S1**.

### Fungal Keratitis Among Patients Clinically Suspected of Microbial Keratitis

**Figure 2:**
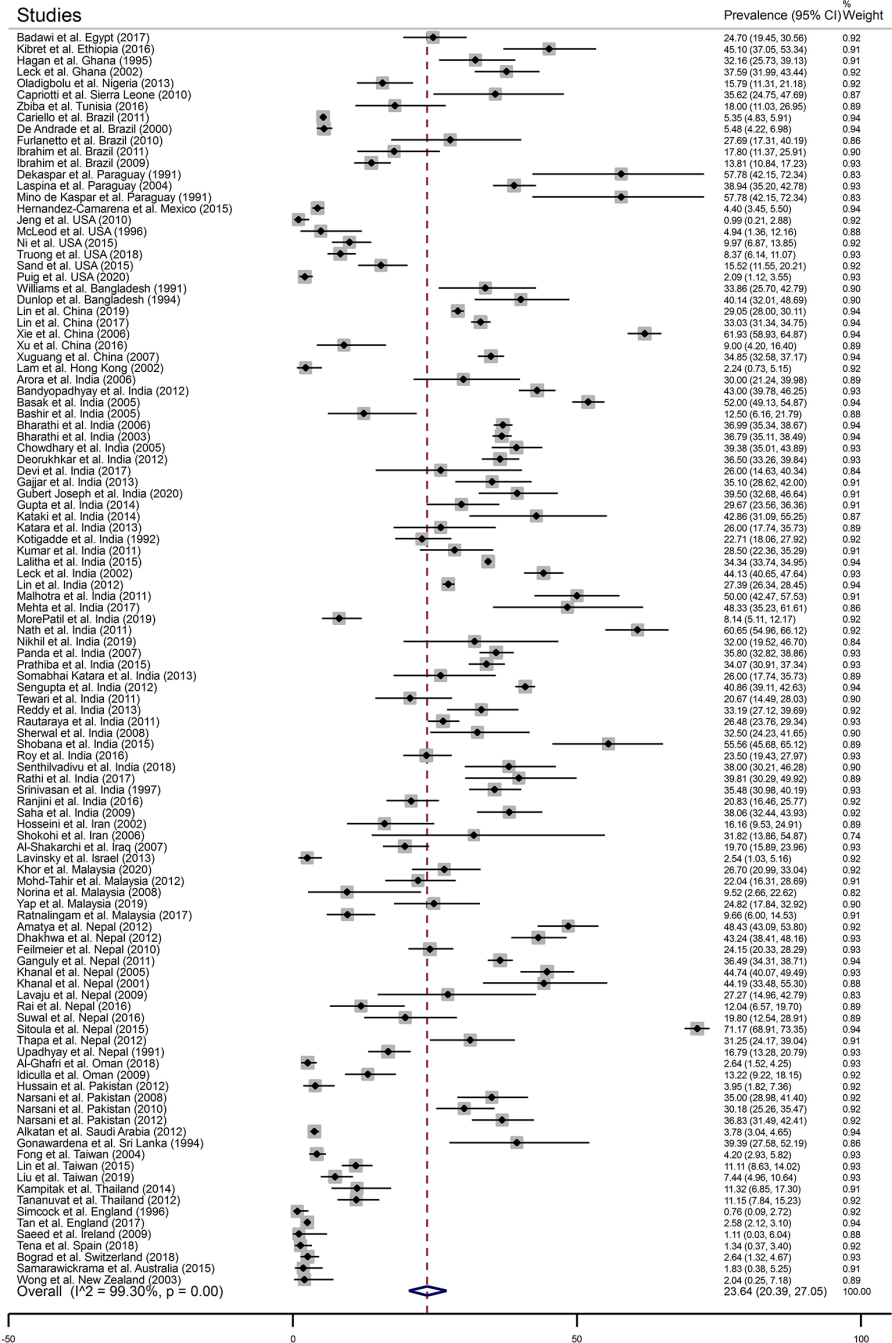
The forest plot of the prevalence of fungal keratitis among patients with a clinical suspicion of microbial keratitis based on the reported articles between January 1, 1990 and May 27, 2020.

In total, 109 articles were included in this group. Based on the analysis, the pooled prevalence of fungal keratitis among these patients was 23.64% (95% CI, 20.39, 27.05) (**Figure 2**), and the prevalence of mold infections was found to be 87.01% (95% CI, 83.31, 90.36) (**Figure S1**). There was no evidence of publication bias among these studies (**Figure S2**). Data of prevalence were available for 31 countries. According to the results of subgroup analysis which are shown in **Table 2**, the highest and the lowest prevalence has been reported from Paraguay [50.06% (95% CI, 35.11, 65.00)] and Ireland [1.11% (95% CI, 0.03, 6.04)], respectively. Based on the results of meta-regression analysis, no significant change was noted in the prevalence over 30 years of study (*p*-value = 0.081) (**Figure S3**). There was also no association between the prevalence and the sample size studied in each report (*p*-value = 0.658) (**Figure S4**).

**Table 2:** The pooled prevalence of fungal keratitis among patients clinically suspected of microbial keratitis in various countries (1990 up to May 27, 2020).

Country	Pooled prevalence	95% confidence interval
Paraguay	50.06	35.11, 65.00
Ethiopia	45.10	37.05, 53.34
Sri Lanka	39.39	27.58, 52.19
Bangladesh	37.15	31.35, 42.95
Sierra Leone	35.62	24.75, 47.69
Ghana	35.35	31.16, 39.66
Nepal	34.42	23.38, 46.37
India	34.18	31.82, 36.58
China	33.18	23.64, 43.46
Egypt	24.70	19.45, 30.56
Pakistan	24.49	10.06, 42.71
Iraq	19.70	15.89, 23.96
Malaysia	18.44	11.48, 26.57
Iran	18.33	11.70, 25.96
Tunisia	18.00	11.03, 26.95
Nigeria	15.79	11.31, 21.18
Brazil	11.60	7.10, 17.01
Thailand	11.19	8.45, 14.25
Taiwan	7.30	3.54, 12.24
USA	6.06	2.33, 11.31
Oman	4.86	3.49, 6.43
Mexico	4.40	3.45, 5.50
Saudi Arabia	3.78	3.04, 4.65
Switzerland	2.64	1.32, 4.67
Israel	2.54	1.03, 5.16
England	2.37	1.94, 2.85
Hong Kong	2.24	0.73, 5.15
New Zealand	2.04	0.25, 7.18
Australia	1.83	0.38, 5.25
Spain	1.34	0.37, 3.40
Ireland	1.11	0.03, 6.04

From a total of 15,295 fungal isolates, 13,048 were identified. These isolates belong to 63 distinct genera, and *Fusarium* species (*n* = 5,294, 40.57%) followed by *Aspergillus* species (*n* = 4,047, 31.02%), *Curvularia* species (*n* = 777, 5.95%), and *Candida* species (*n* = 582, 4.46%) were the most common causes of the disease. As shown in **Table 3**, 628 of 5,294 *Fusarium* isolates were identified at the species level and *Fusarium solani* was the most common species. Similarly, 1,405 of 4,666 *Aspergillus* have been identified at the species level and *Aspergillus flavus* was the most common species. Among *Candida* isolates, 479 have been identified at the species level revealing *Candida albicans* as the most common species.

**Table 3:** The spectrum and frequency of various fungi isolated from patients with suspected microbial keratitis and identified at the genus/species level during 1990 to 2020 (13,048 out of 15,295 isolates have been identified).

Fungus	Frequency (*N* = 13,048)	Percentage	Fungus	Frequency	Percentage
*Fusarium*	5,294	40.57	*Trichophyton*	21	0.16
* Fusarium solani*	361	**6.82**	*Lasiodiplodia*	21	0.16
* Fusarium oxysporum*	136	**2.57**	*Fonsecaea*	12	0.09
* Fusarium moniliforme*	116	**2.19**	*Scopulariopsis*	12	0.09
* Fusarium nivale (Microdocium nivale)*	11	**0.21**	*Rhodotorula*	10	0.08
* Fusarium subglutinans*	3	**0.06**	*Cephalosporium*	10	0.08
* Fusarium verticillioides*	1	**0.02**	*Phialophora*	8	0.06
Unidentified species	4,666	**88.14**	*Hormodendrum*	7	0.05
*Aspergillus*	4,047	31.02	*Colletotrichum*	6	0.05
* Aspergillus flavus*	562	**13.89**	*Trichosporon*	6	0.05
* Aspergillus fumigatus*	487	**12.03**	*Verticillium*	5	0.04
* Aspergillus niger*	330	**8.15**	*Cylindrocarpon*	5	0.04
* Aspergillus terreus*	17	**0.42**	*Drechslera*	4	0.03
* Aspergillus nidulans*	4	**0.10**	*Chaetomium*	4	0.03
* Aspergillus oryzae*	2	**0.05**	*Exophiala*	4	0.03
* Aspergillus versicolor*	2	**0.05**	*Chrysosporium*	4	0.03
* Aspergillus tamarii*	1	**0.02**	*Trichoderma*	3	0.02
Unidentified species	2,642	**65.28**	*Sepedonium*	3	0.02
*Candida*	582	4.46	*Phoma*	3	0.02
* Candida albicans*	395	**67.87**	*Nigrospora*	3	0.02
* Candida parapsilosis*	44	**7.56**	*Syncephalastrum*	3	0.02
* Candida tropicalis*	28	**4.81**	*Geotrichum*	2	0.02
* Candida glabrata*	4	**0.69**	*Epicoccum*	2	0.02
* Candida dubliniensis*	2	**0.34**	*Dichotomophthoropsis*	2	0.02
* Candida rugosa*	2	**0.34**	*Curwlanum*	2	0.02
* Candida krusei*	1	**0.17**	*Dichotomophthoropsis*	2	0.02
* Candida pelliculosa* (*Pichia anomala*)	1	**0.17**	*Diplosporium*	2	0.02
* Candida utilis*	1	**0.17**	*Microsporum*	2	0.02
* Candida guilliermondii*	1	**0.17**	*Monilia*	2	0.02
Unidentified species	103	**17.70**	*Graphium*	2	0.02
*Curvularia*	777	5.95	*Monosporium*	2	0.02
*Penicillium*	389	2.98	*Ustilago*	1	0.01
*Helminthosporium*	332	2.54	*Absidia*	1	0.01
*Mucor*	305	2.34	*Madurella*	1	0.01
*Cladosporium*	230	1.76	*Phaeosiaria*	1	0.01
*Alternaria*	168	1.29	*Cephaliophora*	1	0.01
*Bipolaris*	152	1.16	*Keratomyces ajilloi*	1	0.01
*Botryodiplodia*	143	1.10	*Diplodia*	1	0.01
*Acremonium*	133	1.02	*Gymnoascus*	1	0.01
*Exserohilum*	101	0.77	*Epidermophyton*	1	0.01
*Scedosporium*	56	0.43	*Sporothrix*	1	0.01
*Aureobasidium*	43	0.33	*Cladophialophora*	1	0.01
*Zymoid epiphyte*	41	0.31	*Scytalidium*	1	0.01
*Rhizopus*	35	0.27	*Arthrographis*	1	0.01
*Paecilomyces*	32	0.25	*Cryptococcus*	1	0.01

Numbers in boldface are intragenus percentages of various Fusarium, Aspergillus, and Candida species.

### Fungal Keratitis Among Patients With Culture-Confirmed Microbial Keratitis

Of the 169 articles, 10 met the inclusion criteria to be categorized in this group. These articles studied patients with a positive microbial culture. The pooled prevalence of fungal keratitis in this group was 17.89% (95% CI, 6.96, 32.42) (**Figure S5**), of them, 76.63% (95% CI, 53.16, 94.16) were due to molds (**Figure S6**).

From a total of 2,016 fungal isolates from these patients, 1,636 isolates were identified, mainly at the genus level. As shown in **Table 4**, the identified isolates belong to 27 distinct genera, and *Fusarium* species (*n* = 771, 47.13%) were the most common cause of the disease followed by members of *Aspergillus* (*n* = 584, 35.70%), *Candida* (*n* = 82, 5.01%), and *Curvularia* (*n* = 52, 3.18%).

**Table 4:** The spectrum and frequency of various fungi isolated from patients with confirmed microbial keratitis and identified at the genus level during 1990 to 2020 (1,636 out of 2,016 isolates have been identified).

Fungus	Frequency (*N* = 1,636)	Percentage	Fungus	Frequency	Percentage
*Fusarium*	771	47.13	*Chrysosporium*	6	0.37
*Aspergillus*	584	35.70	*Cryptococcus*	3	0.18
*Candida*	82	5.01	*Rhizopus*	2	0.12
*Curvularia*	52	3.18	*Scytalidium*	2	0.12
*Acremonium*	21	1.28	*Chaetomium*	2	0.12
*Trichophyton*	20	1.22	*Exophiala*	2	0.12
*Bipolaris*	16	0.98	*Rhodotorula*	2	0.12
*Penicillium*	11	0.67	*Scopulariopsis*	1	0.06
*Alternaria*	11	0.67	*Beauveria*	1	0.06
*Exserohilum*	11	0.67	*Colletotrichum*	1	0.06
*Scedosporium*	10	0.61	*Epicoccum*	1	0.06
*Cladosporium*	8	0.49	*Chrysonilia*	1	0.06
*Lasiodiplodia*	7	0.43	*Fonsecaea*	1	0.06
*Paecilomyces*	7	0.43			

### Fungal Keratitis Among Patients Clinically Suspected of Fungal Keratitis

In general, 13 articles were categorized in this group. These articles included studies conducted on patients with a clinical suspicion of fungal keratitis. Thus, those with a clinical suspicion of other types of microbial keratitis have been excluded. Based on the meta-analysis, the pooled estimated prevalence of fungal keratitis among these patients was 43.01% (95% CI, 30.88, 55.59) (**Figure S7**), and in total, 91.76% (95% CI, 87.34, 95.39) were due to molds (**Figure S8**).

From a total of 5,557 isolates from these patients, 5,245 were identified. As shown in **Table 5**, these isolates belong to 42 distinct genera. *Aspergillus* species (*n* = 1,712, 32.64%) were the most common cause of the disease followed by *Fusarium* species (*n* = 1,543, 29.42%), *Curvularia* species (*n* = 480, 9.15), *Alternaria* species (*n* = 478, 9.11%), and *Candida* species (*n* = 283, 5.40%). Two isolates of thermally dimorphic fungi, i.e., *Blastomyces* and *Sporothrix* have also been recovered from these patients.

**Table 5:** The spectrum and frequency of various fungi isolated from patients with clinically suspected fungal keratitis and identified at the genus level during 1990 to 2020 (5,245 out of 5,557 isolates have been identified).

Fungus	Frequency (*N* = 5,245)	Percentage	Fungus	Frequency (*N* = 5,245)	Percentage
*Aspergillus*	1,712	32.64	*Absidia*	2	0.04
*Fusarium*	1,543	29.42	*Exophiala*	2	0.04
*Curvularia*	480	9.15	*Scytalidium*	2	0.04
*Alternaria*	478	9.11	*Epicoccum*	2	0.04
*Candida*	283	5.40	*Acrophialophora*	2	0.04
*Bipolaris*	255	4.86	*Drechslera*	2	0.04
*Penicillium*	197	3.76	*Trichosporon*	2	0.04
*Acremonium*	89	1.70	*Scopulariopsis*	1	0.02
*Rhizopus*	43	0.82	*Cylindrocarpon*	1	0.02
*Paecilomyces*	41	0.78	*Neurospora*	1	0.02
*Rhodotorula*	22	0.42	*Lasiodiplodia*	1	0.02
*Scedosporium*	16	0.31	*Beauveria*	1	0.02
*Exserohilum*	15	0.29	*Phialophora*	1	0.02
*Cladosporium*	12	0.23	*Rhinocladiella*	1	0.02
*Mucor*	7	0.13	*Nigrospora*	1	0.02
*Fonsecaea*	6	0.11	*Papulaspora*	1	0.02
*Aureobasidium*	5	0.09	*Sarcinomyces*	1	0.02
*Chaetomium*	4	0.08	*Trichoderma*	1	0.02
*Trichophyton*	4	0.08	*Microsporum*	1	0.02
*Colletotrichum*	3	0.06	*Blastomyces*	1	0.02
*Chrysosporium*	2	0.04	*Sporothrix*	1	0.02

### Fungal Keratitis Among Pediatric Patients

Studies categorized in this group have been done on patients aged ≤16 years, except for one study which was done on patients ≤15 years with a clinical and/or microbiological diagnosis of microbial keratitis (nonviral). Using this criterion, eight articles were identified. Due to difference in inclusion criteria from articles grouped as clinically suspected microbial, culture-confirmed microbial, and clinically suspected fungal, these studies and the next two groups (contact lens wearers and postkeratoplasty patients) were analyzed separately. The pooled estimated prevalence of fungal keratitis among these patients was 14.88% (95% CI, 6.87, 25.11) (**Figure S9**) and molds accounted for 95.30% (95% CI, 84.10, 100.00) of cases (**Figure S10**). In total, 163 isolates were recovered from these patients, of them, 141 were identified (**Table 6**). These isolates belong to seven genera; *Fusarium* species (*n* = 88, 62.41%) followed by *Aspergillus* species (*n* = 31, 21.99%) were the most common causes.

**Table 6:** The spectrum and frequency of various fungi isolated from pediatric patients with fungal keratitis and identified at the genus level during 1990 to 2020 (141 out of 163 isolates have been identified).

Fungus	Frequency (*N* = 141)	Percentage
*Fusarium*	88	62.41
*Aspergillus*	31	21.99
*Candida*	16	11.35
*Curvularia*	3	2.13
*Acremonium*	1	0.71
*Alternaria*	1	0.71
*Bipolaris*	1	0.71

### Fungal Keratitis Among Contact Lens Wearers

Studies categorized in this group were those that reported the prevalence of fungal keratitis among patients with a history of nontherapeutic contact lens use (e.g., cosmetic contact lenses). Using the inclusion criteria, six articles were identified, and based on the meta-analysis, the pooled estimated prevalence of fungal keratitis among them was 18.05% (95% CI, 1.04, 46.91) (**Figure S11**). Almost all the cases (37 out of 38) were due to molds, and the pooled estimated prevalence of mold infections was 100% (95% CI, 94.30, 100) (**Figure S12**).

From 38 fungal isolates, 35 were identified including *Fusarium* species (*n* = 31, 88.57%), *Aspergillus* species (*n* = 3, 7.89%), and *Acremonium* species (*n* = 1, 2.63%).

### Fungal Keratitis Among Postkeratoplasty Patients

Twenty-three articles met the inclusion criteria that were focused on patients who had undergone keratoplasty. We divided these articles into two groups, seven articles that provided data on patients who had undergone keratoplasty due to a variety of indications except infective keratitis, and 16 articles that provided data on patients who had undergone keratoplasty due to infective keratitis. This was done because the denominator of the latter group was smaller than that of the former. The pooled prevalence of fungal keratitis (reinfection or recurrence) was 0.05% (95% CI, 0.00, 0.14) and 8.57% (95% CI, 3.89, 14.62) in these groups, respectively (**Figure S13**). In general, the prevalence of yeast was higher among these patients with a pooled value of 51.80% (95% CI, 14.41, 88.30) (**Figure S14**).

Data of the causative fungi were available only in 13 articles. From the total of 379 isolates, 294 (77.57%) were identified. As shown in **Table 7**, these isolates belonged to 13 distinct genera, including one isolate of *Pythium*, a member of Oomycota (not true fungi). *Fusarium* species (*n* = 124, 42.18%) were the dominant cause followed by *Aspergillus* (*n* = 81, 27.55%) and *Candida* (*n* = 60, 20.41%) species. Among 27 isolates of *Aspergillus* that were identified to the species level, 22, three, and two isolates were found to be *A. flavus*, *A. niger*, and *A. fumigatus*, respectively.

**Table 7:** The spectrum and frequency of various fungi isolated from keratoplasty patients with fungal keratitis and identified at the genus level during 1990 to 2020 (294 out of 379 isolates have been identified).

Fungus	Frequency (*N* = 294)	Percentage
*Fusarium*	124	42.18
*Aspergillus*	81	27.55
*Candida*	60	20.41
*Curvularia*	6	2.04
*Alternaria*	5	1.70
*Acremonium*	4	1.36
*Penicillium*	3	1.02
*Phythium* ^a^	3	1.02
*Scedosporium*	2	0.68
*Bipolaris*	2	0.68
*Paecilomyces*	2	0.68
*Trichosporon*	1	0.34
*Wangiella*	1	0.34

a A member of Oomycota (not true fungi).

### Mixed Fungal and Bacterial Keratitis

The prevalence of mixed fungal and bacterial keratitis was calculated regardless of the grouping schedule. In this calculation, articles reporting data on keratoplasty recipients and patients with suspected fungal keratitis were excluded because the data of mixed infections were not available in almost all of these articles. From the remaining four article groups, data of mixed infections were extractable in 110 articles. Based on these analyses, the pooled prevalence of mixed infections was 9.29% (95% CI, 6.52, 12.38).

## Discussion

A systematic assessment of the 169 articles from 36 countries (**Table S1**) revealed the geographic variation in the prevalence rates and predominant etiological genera of FK. The epidemiological patterns of FK can differ from one country to the other, as well as in different geographical regions of the same country (Mahmoudi et al., 2018).

In the present study, the majority of large-scale investigations were reported from India, China, the USA, Nepal, Taiwan, and Brazil in descending rank. In contrast, fewer studies were reported from Europe and Oceania continents where climatic conditions and nonagricultural activities result in low frequency of cases in these countries.

Given that, the highest pooled prevalence of FK were recorded in countries such as Paraguay, Ethiopia, Sri Lanka, and Bangladesh (**Table 2**), which had the lowest eligible studies for inclusion in our survey (**Table S1**); these prevalence rates might be less reliable than those documented in countries with high eligible studies such as India, China, USA, Nepal, Taiwan, and Brazil (**Table 2** and **Table S1**).

The general consensus is that FK is more frequent in developing countries within tropical and subtropical climate in comparison with developed countries having cold or temperate climate (Kredics et al., 2015; Mahmoudi et al., 2018). Evidence is strong that the prevalence of FK is highly favored in areas with a warm, humid climate and an agricultural economy and its frequency has been estimated to range from 20% to 60% of all culture-positive corneal infections in these regions (Brown et al., 2020).

The comprehensive data, particularly the prevalence rates and predominant causal agents of FK in different regions and patient populations, are indispensable to having appropriate diagnostic and therapeutic strategies (Kredics et al., 2015; Mahmoudi et al., 2018). Nonetheless, there is no systematic survey estimating the rate of fungal keratitis in different population-based studies. This systematic review provides a global frequency of FK in different patient groups and countries. The prevalence ratios given for FK depend on the settings and the population under study, thus it should be carefully compared, because of the different inclusion criteria used to select the studied subjects and the variations in sensitivity of the modalities used for FK diagnosis (Brown et al., 2020). Depending on the populations examined in the present study, the prevalence of FK reported was up to 43% (95% CI, 0.05–43%). The highest and lowest prevalence of FK were documented in patients with a clinical suspicion of keratomycosis and those who underwent keratoplasty, respectively (43% *vs*. 0.05%). The prevalence of FK in the keratomycosis suspected group was anticipated to be higher in comparison with suspected-microbial keratitis patients (43% *vs*. 23.6%). This is attributable to higher proportion of cases that were clinically suspected to have FK than in studies where ocular infections might have been caused by any of the fungal, bacterial, viral, amoebic, oomycete, or parasitic agents (Cariello et al., 2011). Similarly, previous large-scale retrospective analyses investigated in India, Turkey, Paraguay, and Brazil reported much lower rates of FK (23%, 22.3%, 20.6%, and 5.3%, respectively) in corneal cultures obtained from patients with a suspicion of microbial keratitis (Laspina et al., 2004; Yilmaz et al., 2007; Gopinathan et al., 2009; Cariello et al., 2011). However, regardless of variable frequency of FK in different geographical locations, the low sensitivity of traditional microbiological methods (culture, smear stains) routinely used to diagnose microbial keratitis is accounted for by the fact that many of the FK-affected people are the resident of rural areas and might never visit medical centers because of low income, high cost of treatment, and long distance. The variable frequency of FK may also be due to the fact that most studies conducted at tertiary healthcare facilities, accepting more severe cases, resulting in overestimation of FK, which makes the assessment of real prevalence and incidence rate of FK more complicated.

Prevalence surveys done on patients with culture-confirmed microbial keratitis determined that 17.9% of microbial keratitis cases were caused by FK that was lower than both above mentioned groups. Considering the limitation of culture-based methods (Mahmoudi et al., 2018), the lower rate of FK frequency documented in surveys including only culture-positive specimens is expected. Although culture methods remained the cornerstone of FK diagnosis in most studies (Kredics et al., 2015), the isolation rates differed among different studies and the sensitivity of culture was as low as 50% (Fong et al., 2004). It is also known that culture-negative cases of FK may show fungal filaments in microscopic examination of the corneal scrapings and can be diagnosed as FK anyway (Sharma et al., 2002). Empirical treatment with topical antibiotics, use of topical anesthetics with potential antimicrobial effects, different methods of sample collections (swabbing *vs*. corneal scraping), the low quantity of specimens available for culture, refractory nature of fungi, and debatable adverse effect of transport devices or media on the viability of microorganisms, are the possible confounders which may influence the results of culture method (Fong et al., 2004; Kredics et al., 2015; Mahmoudi et al., 2018).

In the contact lens wearer (CLW) group, the pooled prevalence of FK was found to be 18.05%. In 25% to 40% of keratomycosis cases particularly those living in developed countries, CL wear is evidenced as a risk factor (Gopinathan et al., 2009; Bourcier et al., 2017). Corneal defects, gradually caused by CL wear, poor hygiene practices such as overnight wear of CL, and ineffective or contaminated cleaning solution, are within the list of factors increasingly associated with fungal keratitis in CLWs (Mahmoudi et al., 2018; Brown et al., 2020). CL-associated FK has primarily occurred in individuals with low socioeconomic status in which poor knowledge about hygienic eye care and inadequate cleaning solution are attributable risk factors (Mahmoudi et al., 2018; Brown et al., 2020).

In the pediatric setting, the prevalence of FK was low (14.9%). Generally, men with agricultural and outdoor occupations and those aged 20–50 years, form a greater proportion of the FK-affected population and are more susceptible than women to develop mycotic keratitis (Gopinathan et al., 2009; Kredics et al., 2015; Bourcier et al., 2017; Mahmoudi et al., 2018; Brown et al., 2020). Nonetheless, children constitute about 4% of keratomycosis cases (Brown et al., 2020). Children have minimal encounters with traumatizing agents (plant and animal sources), which are frequently associated with FK (Brown et al., 2020). One study from southern California reported that pediatric keratitis composed 11% of all cases with microbial keratitis (Ormerod et al., 1986). Another retrospective study from Taiwan reported that pediatric keratitis constituted 13.1% of all cases of infectious keratitis. Meanwhile, the frequency of pediatric fungal keratitis was as low as 6.4% among all culture-positive pediatric microbial keratitis in Taiwanese children (Hsiao et al., 2007).

The rate of FK was 0.05% and 8.5% postkeratoplasty when the relevant studies were divided into two groups, indicating the higher rate of prevalence in the population who underwent keratoplasty after infectious keratitis. It has been demonstrated that the relative risk of fungal keratitis occurrence following corneal allograft is three times elevated in comparison with bacterial infection when a cornea is maintained more than 4 days in preservation medium. Therefore supplementation of donor preservation media with an antifungal agent may be necessary (Hassan et al., 2005). Postkeratoplasty FK in transplant recipients is primarily associated with infection of donor corneal tissue (Aldave et al., 2013). However, the higher rate of FK in populations who underwent keratoplasty after infectious keratitis was postulated to be unlinked to the donor corneal tissue and was probably related to the re-emergence of untreated or partially treated FK.

Although the infection is commonly rated to be rare (Aldave et al., 2013), many ophthalmic surgeons believe that the incidence of postkeratoplasty fungal infections is rising. Fungal infection postendothelial keratoplasty (EK) is thought to be more frequent than penetrating keratoplasty (PK) (Aldave et al., 2013). The Eye Bank Association of America reviewed the frequency of keratoplasty-associated fungal infections and found that the development of fungal infections occurs in 0.052% of anterior lamellar keratoplasty procedures, 0.022% of EK procedures, and 0.012% of PK procedures (Aldave et al., 2013).

Given the variations in antifungal susceptibility patterns of different fungal genera and even different species belonging to the same genus, definitive identification of the etiology to the species level is recommended (Kredics et al., 2015; Garg et al., 2016; Bourcier et al., 2017; Mahmoudi et al., 2018). Nevertheless, in some studies, the nature of agents causing fungal infection was determined only by histopathology (Kredics et al., 2015). On the other hand, the majority of early studies reporting the epidemiology of FK have resorted to the identification of causative agents solely through culture-based morphologies, such that the identification was limited to the genus level (Kredics et al., 2015; Mahmoudi et al., 2018). In the current review, we found the highest (94%) and lowest (77.5%) percentages of fungal identification (principally to the genus level) in studies done on cases with FK suspicion and keratoplasty patients, respectively. Consequently, the identification of the isolates to the species level has been provided in only a few reports. More so, species identification is primarily achieved through morphology-based methods which may lead to a delayed or erroneous diagnosis and misidentification in a significant number of cases.

The subjective morphology-based speciation is prone to be affected by the expertise of the investigator. Investigation using molecular-based methods has been less frequently applied for species identification, which resulted in many causative agents, especially the non-sporulating molds to remain unidentified (Kredics et al., 2015; Mahmoudi et al., 2018). Since, a wide variety of fungal agents are known to be implicated in keratitis (Gopinathan et al., 2009; Kredics et al., 2015; Mahmoudi et al., 2018; Brown et al., 2020), considerable mycological facilities, skills, and expertise are required for reliable identification of culture-positive cases and to rule out contaminants. In those groups of studies that provided molecular based-identification, the internal transcribed spacer (ITS) region of the ribosomal RNA gene cluster (rDNA) was the locus most frequently targeted for species identification. This revealed the highest likelihood of successful delineation for the broadest range of fungi when compared with other DNA regions used as potential DNA barcode markers for fungi (Kredics et al., 2015). However, there is a difficulty with ITS-based identification of some filamentous fungal associated keratitis. The sequencing of the ITS region in relation to some species belonging to *Fusarium* and *Aspergillus* is not discriminative enough to reveal a precise species identification (Kredics et al., 2015). For example, the majority of studies that applied morphological or ITS sequencing-based identification reported *Fusarium solani* as the predominantly encountered agent of *Fusarium*-associated FK. Nonetheless, partial sequencing of elongation factor-1 (TEF-1α) regions of *F. solani*-associated FK isolates showed the predominance of other cryptic members (*F. keratoplastcum* and *F. falciforme*) of *Fusarium solani* species complex (FSSC) (Tupaki-Sreepurna et al., 2017). Therefore, not only molecular identification of the agents causing FK are of great importance but a precise selection of appropriate gene target providing proper identification can contribute to a better understanding of epidemiologic patterns. However, as documented in our systematic surveys, molecular identification had been only performed in the minority of the included studies.

The prevailing causal pathogens may vary in different geographical locations highlighting the need to know the local epidemiology (Gopinathan et al., 2009; Kredics et al., 2015; Mahmoudi et al., 2018; Brown et al., 2020). Similar to most of the studies across the globe (Höfling-Lima et al., 2005; Gopinathan et al., 2009; Wang et al., 2009), in almost all of our population-based studies (except those done on patients with suspected FK), *Fusarium* species were the most predominant etiology of the disease followed by species belonging to *Aspergillus*, *Candida*, and *Curvularia* genera, which stand as other frequent causes of FK (with a slight difference) in some studies. However, in tropical countries, southern US, Mexico, Central America, South America, Africa, Middle East, China, India, and Southeast Asia, FK occurs mostly from filamentous fungi (particularly fusaria and aspergilli) and are frequently associated with plant material related corneal trauma, outdoor occupations and CLU (Kredics et al., 2015; Bourcier et al., 2017; Mahmoudi et al., 2018). Conversely, yeast associated mycotic keratitis (primarily due to *Candida* or *Cryptococcus*) were observed primarily in temperate countries, Europe and northern USA (Kredics et al., 2015; Bourcier et al., 2017; Mahmoudi et al., 2018), and their infection is mostly linked to factors compromising the immunity of the eye such as local or systemic immunosuppressive agents used for corneal grafts and keratoplasty (Aldave et al., 2013; Kredics et al., 2015; Bourcier et al., 2017; Mahmoudi et al., 2018). Consistent with this observation, in the present study, the highest percentage of *Candida*-associated keratitis (20.41%) was found in the group of patients undergoing keratoplasty (**Table 7**).

*Fusarium* species are a serious threat to vision, especially for those wearing CL. Consistently, the highest rate of *Fusarium* (88.5% and 62.4%) was revealed in studies done on CLWs and pediatric patients, respectively. The popularity of CL wear is the leading factor predisposing these groups of patients to develop FK particularly with *Fusarium* etiology (Fong et al., 2004; Hsiao et al., 2007). The increasing prevalence of *Fusarium* keratitis was concurrently associated with a rising incidence of CL wear (Hsiao et al., 2007; Yu et al., 2007). Interestingly, filamentous fungi (primarily *Fusarium* species) were almost the sole agents causing keratitis in the CL wearer group in the current study. A similar resurgence of exclusive CLU-associated *Fusarium* keratitis was noted in literatures from Hong Kong, Singapore, and the USA (Khor et al., 2006; Gorscak et al., 2007; Yu et al., 2007). CLU has evolved as an important risk factor for *Fusarium* keratitis (Gorscak et al., 2007).

Other than filamentous and yeast fungi, the dimorphic fungi are the third group scarcely reported to cause FK (Bourcier et al., 2017). In our review, we observed two cases of FK due to *Blastomyces* and *Sporothrix* among a wide variety of fungal agents causing keratomycosis in the population with suspected FK. Identification of the rarely reported fungi in this group of studies may be due to the increasing utilization of molecular-based methods for fungal identification, better mycological skill, and expertise of the researchers conducting these studies. In absence of application of molecular techniques, in some of the groups included in this study, the fungal isolates may have remained unidentified.

## Conclusion

This review has illustrated the pooled prevalence of FK in different patient groups. The highest prevalence was demonstrated in the group of studies done on patients with suspected FK. Epidemiological variations within different countries are seen partly because of climatic situations and more so due to the occupation of the population. Filamentous fungi such as *Fusarium* and *Aspergillus* continue to be the most frequently encountered genera in mycotic keratitis, and tend to be predominant in traumatized eyes, CLU, and pediatric groups. The highest rate of *Candida* species was recorded in patients with keratoplasty. Our data showed that the majority of the studies have used a culture-based method for the identification of causal agents up to the genus level and PCR-based identification methods have been infrequently employed. As a result, species-specific therapy is hampered, particularly in cases of less susceptible or resistant species of fungi.

## Author Contributions

The study was designed and supervised by SM. KA, SA, BF, MN, SR, and MG participated in database searching, screening the articles, and data extraction. Quality assessment was performed by MG and SM. PM was in charge of meta-analysis. KA and SM drafted the manuscript. MG and SS participated in drafting the manuscript. SS and SM were in charge of the critical review of the draft. All authors contributed to the article and approved the submitted version.

## Conflict of Interest

The authors declare that the research was conducted in the absence of any commercial or financial relationships that could be construed as a potential conflict of interest.

## Publisher’s Note

All claims expressed in this article are solely those of the authors and do not necessarily represent those of their affiliated organizations, or those of the publisher, the editors and the reviewers. Any product that may be evaluated in this article, or claim that may be made by its manufacturer, is not guaranteed or endorsed by the publisher.

## Supplementary Material

Supplementary Figure 1 The forest plot of the prevalence of yeast and mold keratitis among patients with a clinical suspicion of microbial keratitis based on the reported articles between January 1, 1990 and May 27, 2020.

Supplementary Figure 2 The funnel plot of available studies reporting data on the prevalence of fungal keratitis among patients with a clinical suspicion of microbial keratitis between January 1, 1990 and May 27, 2020 (each circle is representative of one study).

Supplementary Figure 3 Univariate meta-regression analysis of the association between the year of publication and the heterogeneity. Each circle is representative of one study, and its size shows the weight of observed effect sizes. The 95% confidence intervals of the regression line are shown by error bars.

Supplementary Figure 4 Univariate meta-regression analysis of the association between the sample size and the heterogeneity. Each circle is representative of one study, and its size shows the weight of observed effect sizes. The 95% confidence intervals of the regression line are shown by error bars.

Supplementary Figure 5 The forest plot of the prevalence of fungal keratitis among patients with culture-confirmed microbial keratitis based on the reported articles between January 1, 1990 and May 27, 2020.

Supplementary Figure 6 The forest plot of the prevalence of yeast and mold keratitis among patients with culture-confirmed microbial keratitis based on the reported articles between January 1, 1990 and May 27, 2020.

Supplementary Figure 7 The forest plot of the prevalence of fungal keratitis among patients with clinical suspicion of fungal keratitis based on the reported articles between January 1, 1990 and May 27, 2020.

Supplementary Figure 8 The forest plot of the prevalence of yeast and mold keratitis among patients with clinical suspicion of fungal keratitis based on the reported articles between January 1, 1990 and May 27, 2020.

Supplementary Figure 9 The forest plot of the prevalence of fungal keratitis among pediatric patients based on the reported articles between January 1, 1990 and May 27, 2020.

Supplementary Figure 10 The forest plot of the prevalence of yeast and mold keratitis among pediatric patients based on the reported articles between January 1, 1990 and May 27, 2020.

Supplementary Figure 11 The forest plot of the prevalence of fungal keratitis among patients with contact lens wearers based on the reported articles between January 1, 1990 and May 27, 2020.

Supplementary Figure 12 The forest plot of the prevalence of yeast and mold keratitis among contact lens wearers based on the reported articles between January 1, 1990 and May 27, 2020.

Supplementary Figure 13 The forest plot of the prevalence of fungal keratitis among patients undergone keratoplasty based on the reported articles between January 1, 1990 and May 27, 2020.

Supplementary Figure 14 The forest plot of the prevalence of yeast and mold keratitis among patients undergone keratoplasty based on the reported articles between January 1, 1990 and May 27, 2020.
