# Supplementary figures and images for: Distribution, Prevalence, and Causative Agents of Fungal Keratitis: A Systematic Review and Meta-Analysis (1990 to 2020)
Source: Front Cell Infect Microbiol. 2021 Aug 26;11:698780. doi: 10.3389/fcimb.2021.698780 (PMC8428535; doi:10.3389/fcimb.2021.698780)

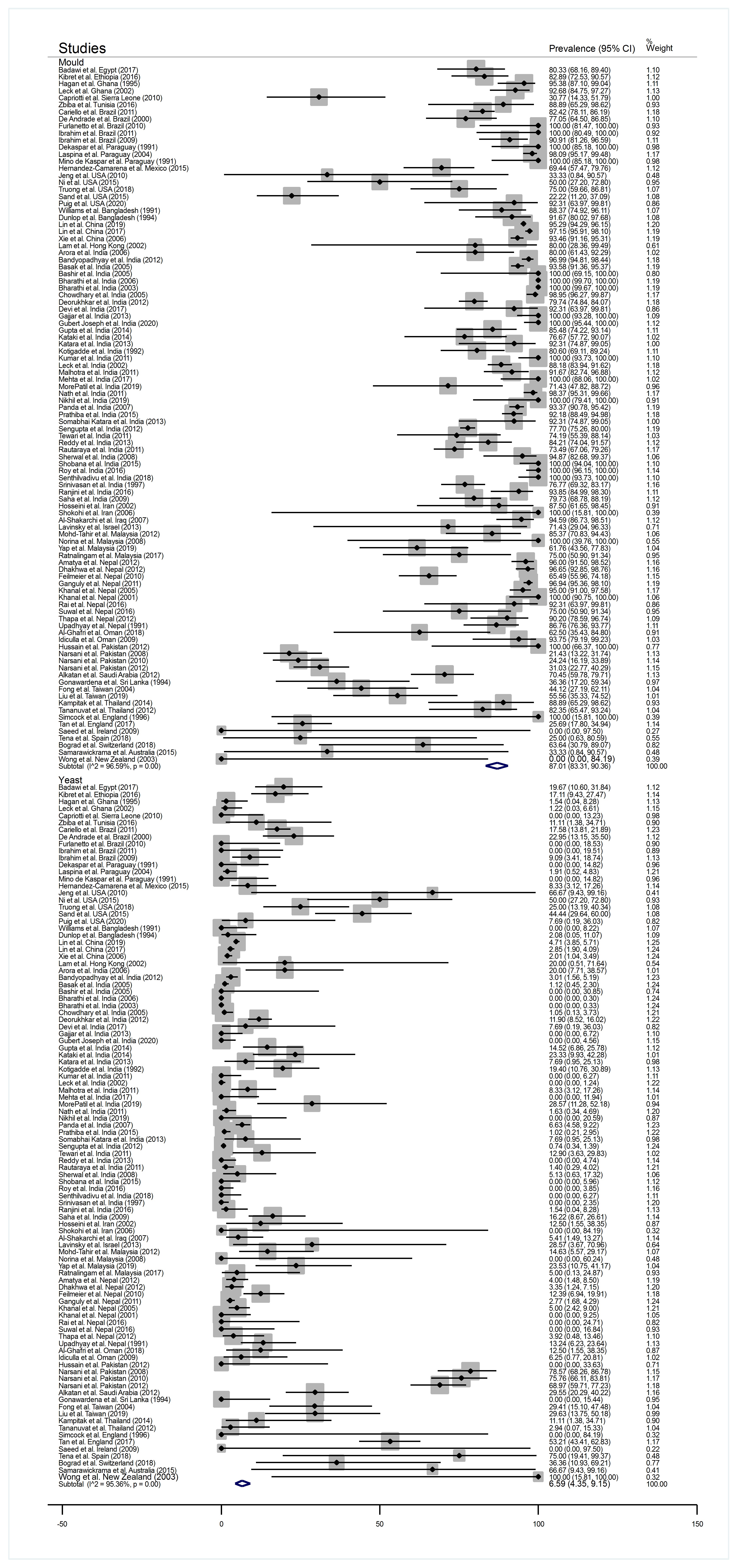

Supplement: Supplementary Figure 1 — The forest plot of the prevalence of yeast and mold keratitis among patients with a clinical suspicion of microbial keratitis based on the reported articles between January 1, 1990 and May 27, 2020. [file Image_1.jpeg]

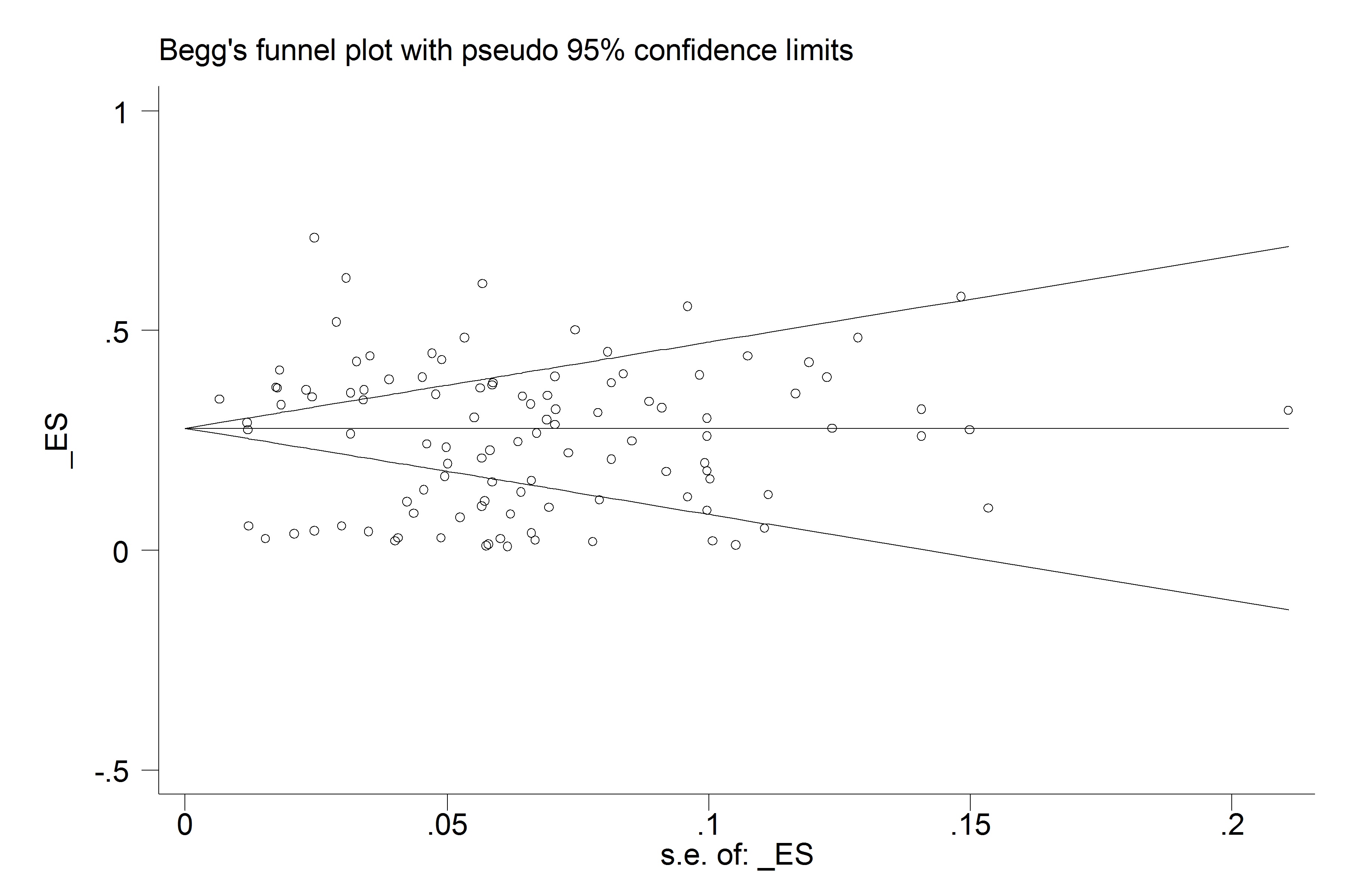

Supplement: Supplementary Figure 2 — The funnel plot of available studies reporting data on the prevalence of fungal keratitis among patients with a clinical suspicion of microbial keratitis between January 1, 1990 and May 27, 2020 (each circle is representative of one study). [file Image_2.jpeg]

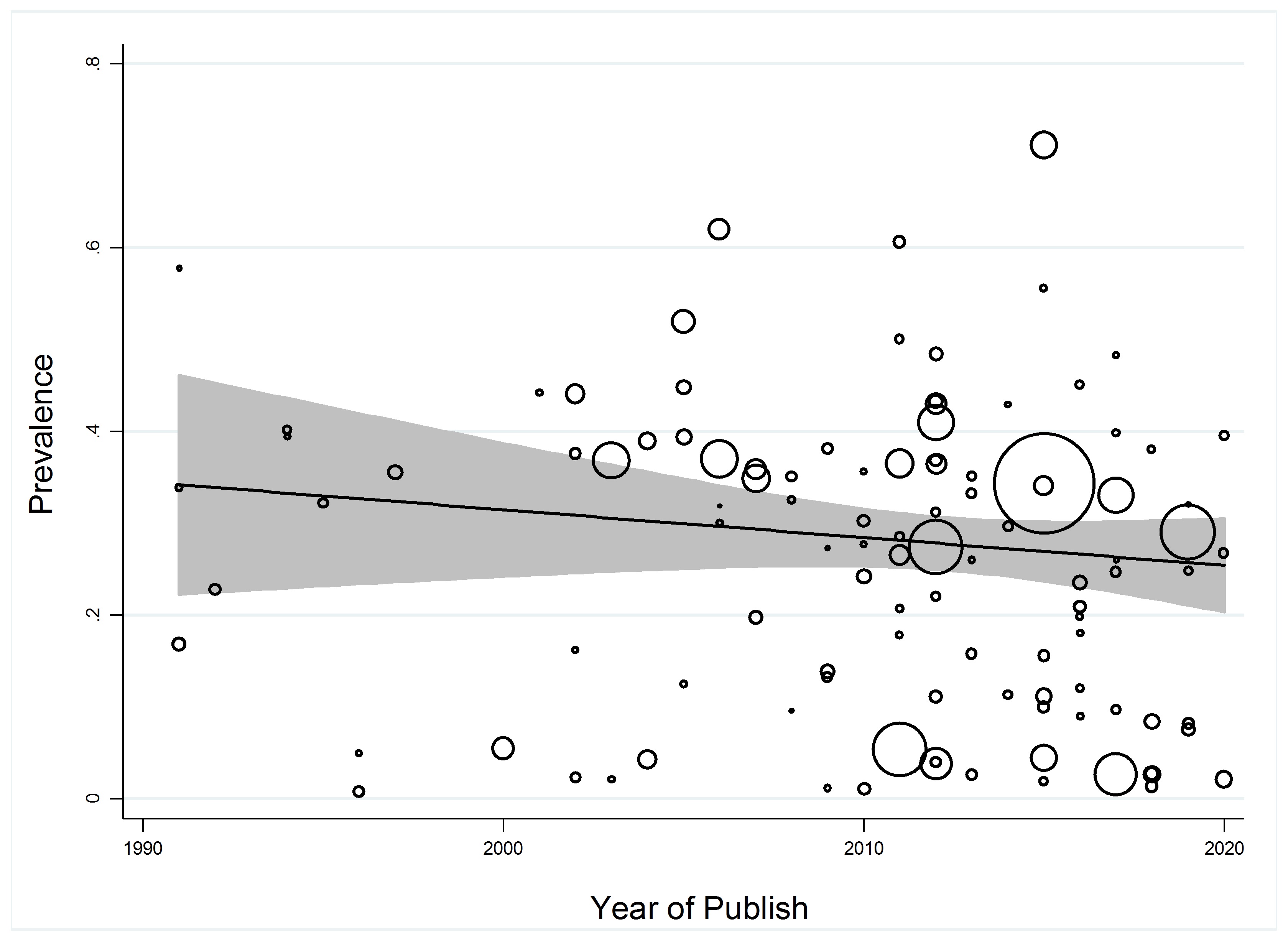

Supplement: Supplementary Figure 3 — Univariate meta-regression analysis of the association between the year of publication and the heterogeneity. Each circle is representative of one study, and its size shows the weight of observed effect sizes. The 95% confidence intervals of the regression line are shown by error bars. [file Image_3.jpeg]

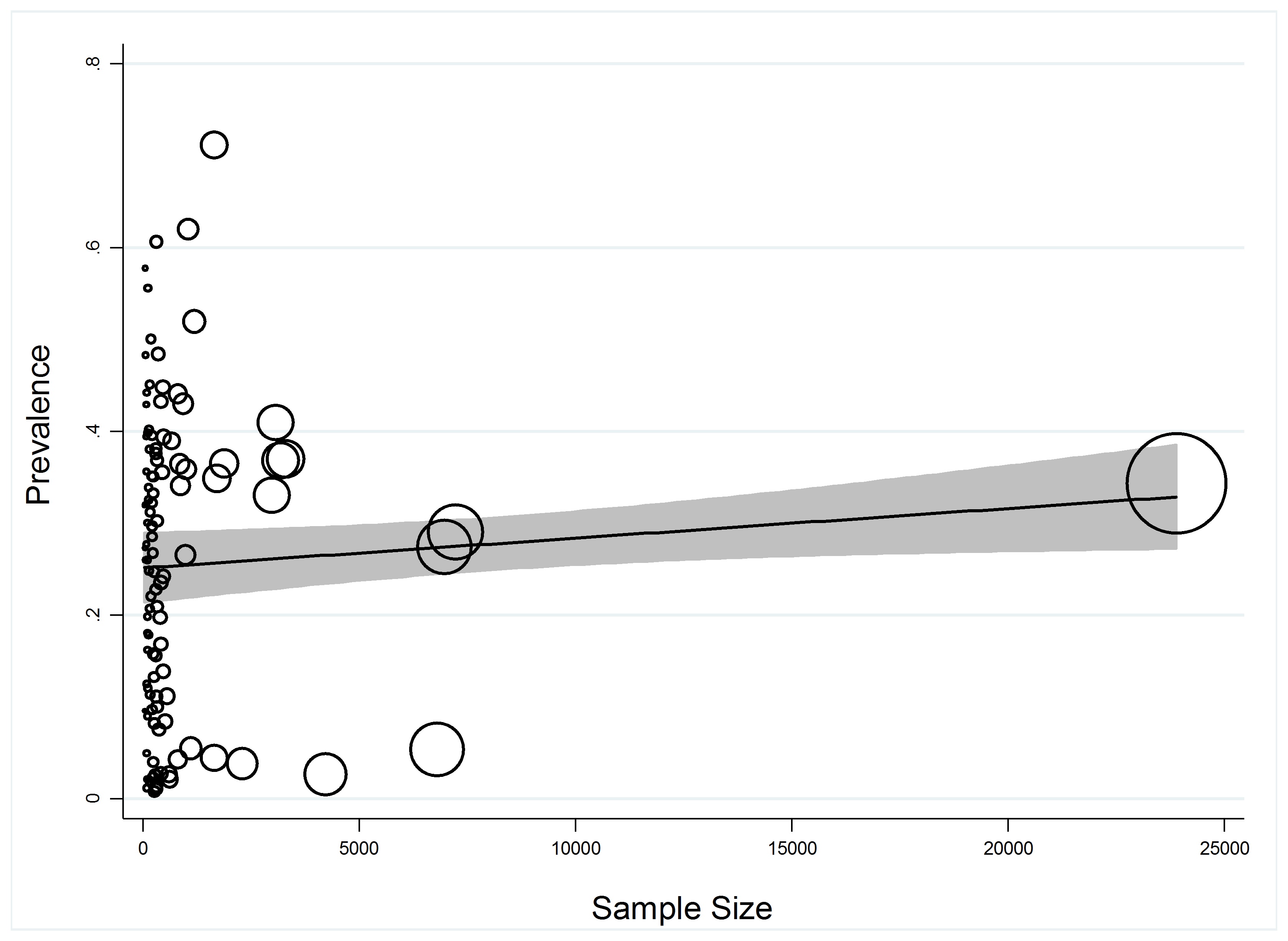

Supplement: Supplementary Figure 4 — Univariate meta-regression analysis of the association between the sample size and the heterogeneity. Each circle is representative of one study, and its size shows the weight of observed effect sizes. The 95% confidence intervals of the regression line are shown by error bars. [file Image_4.jpeg]

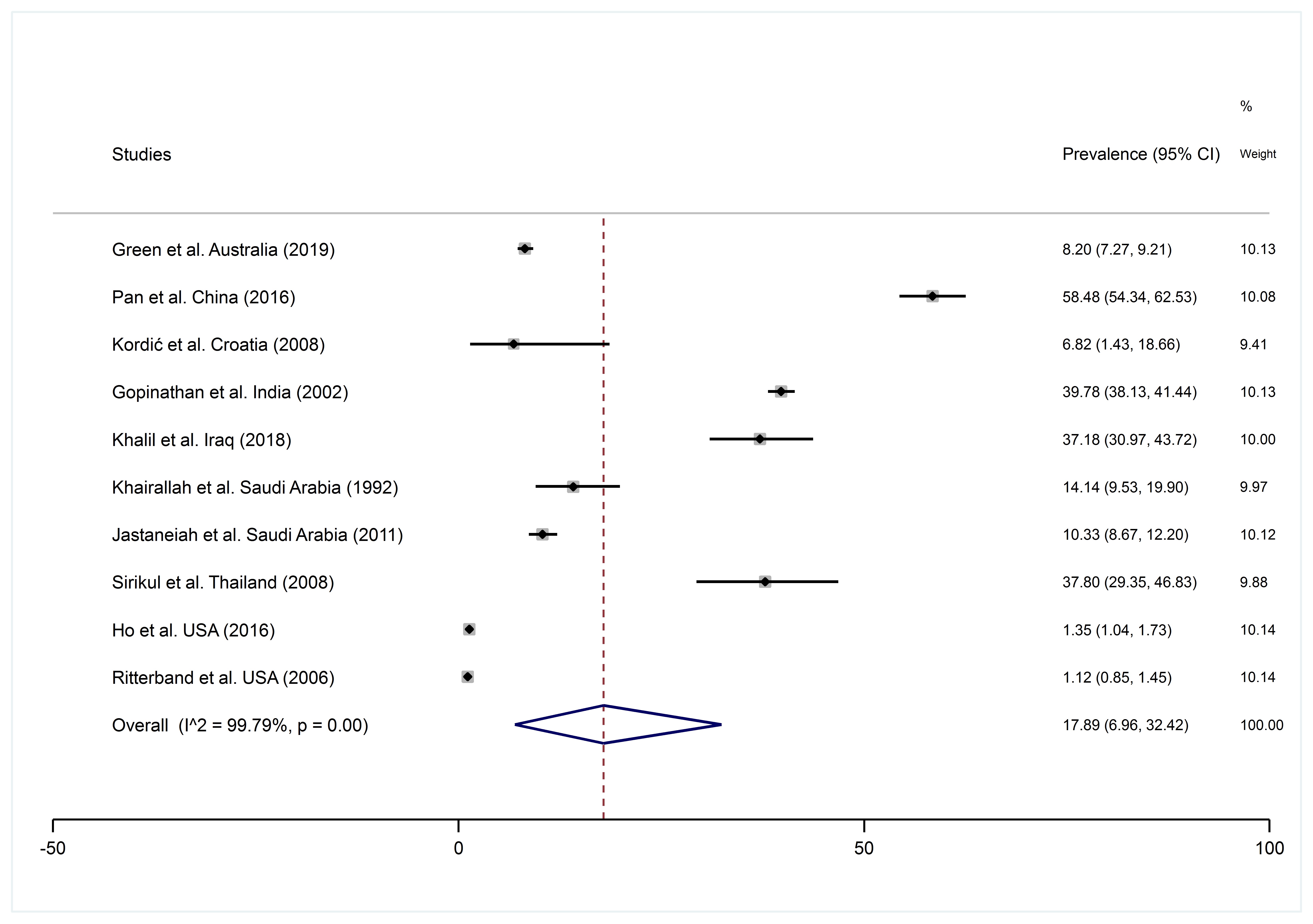

Supplement: Supplementary Figure 5 — The forest plot of the prevalence of fungal keratitis among patients with culture-confirmed microbial keratitis based on the reported articles between January 1, 1990 and May 27, 2020. [file Image_5.jpeg]

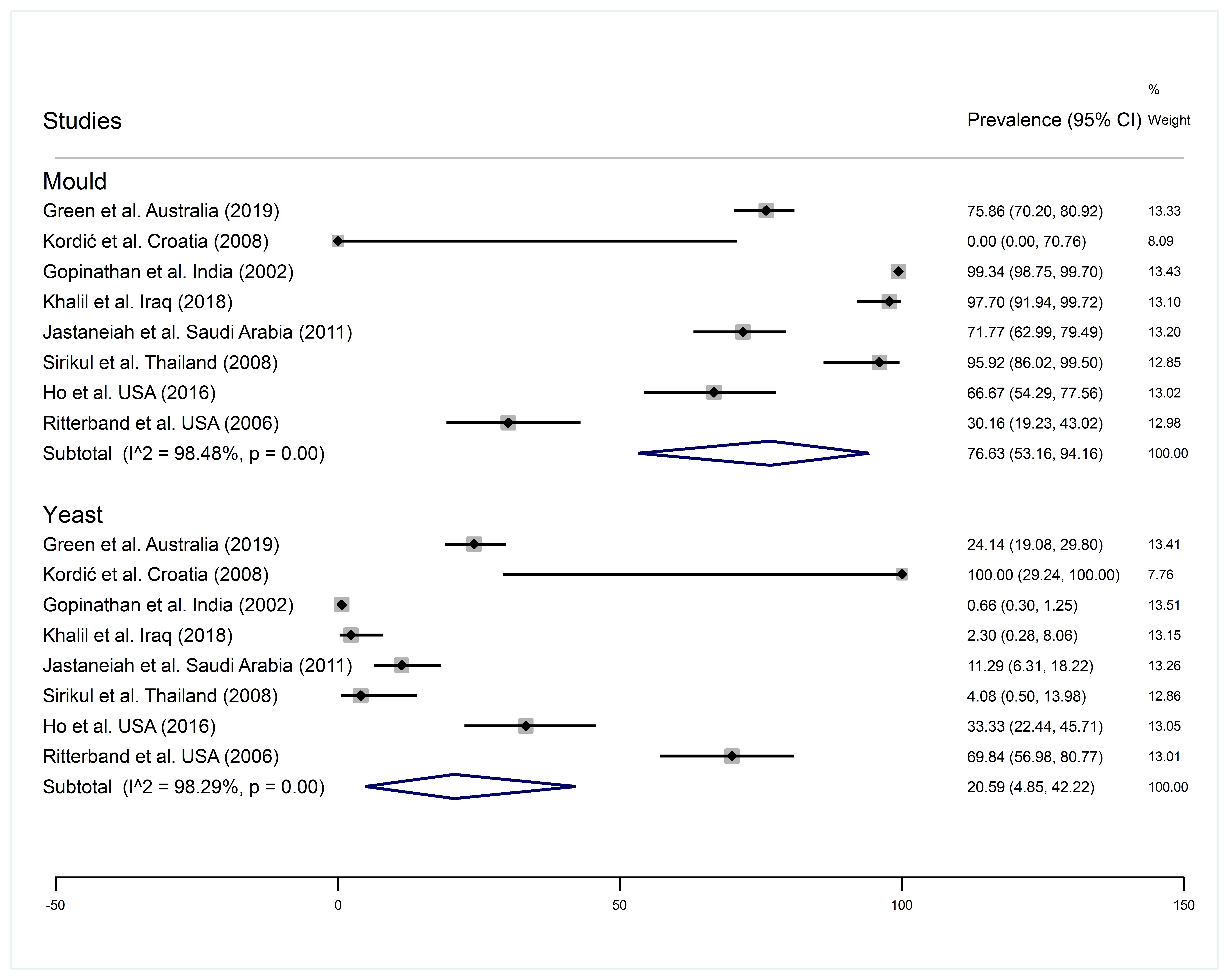

Supplement: Supplementary Figure 6 — The forest plot of the prevalence of yeast and mold keratitis among patients with culture-confirmed microbial keratitis based on the reported articles between January 1, 1990 and May 27, 2020. [file Image_6.jpeg]

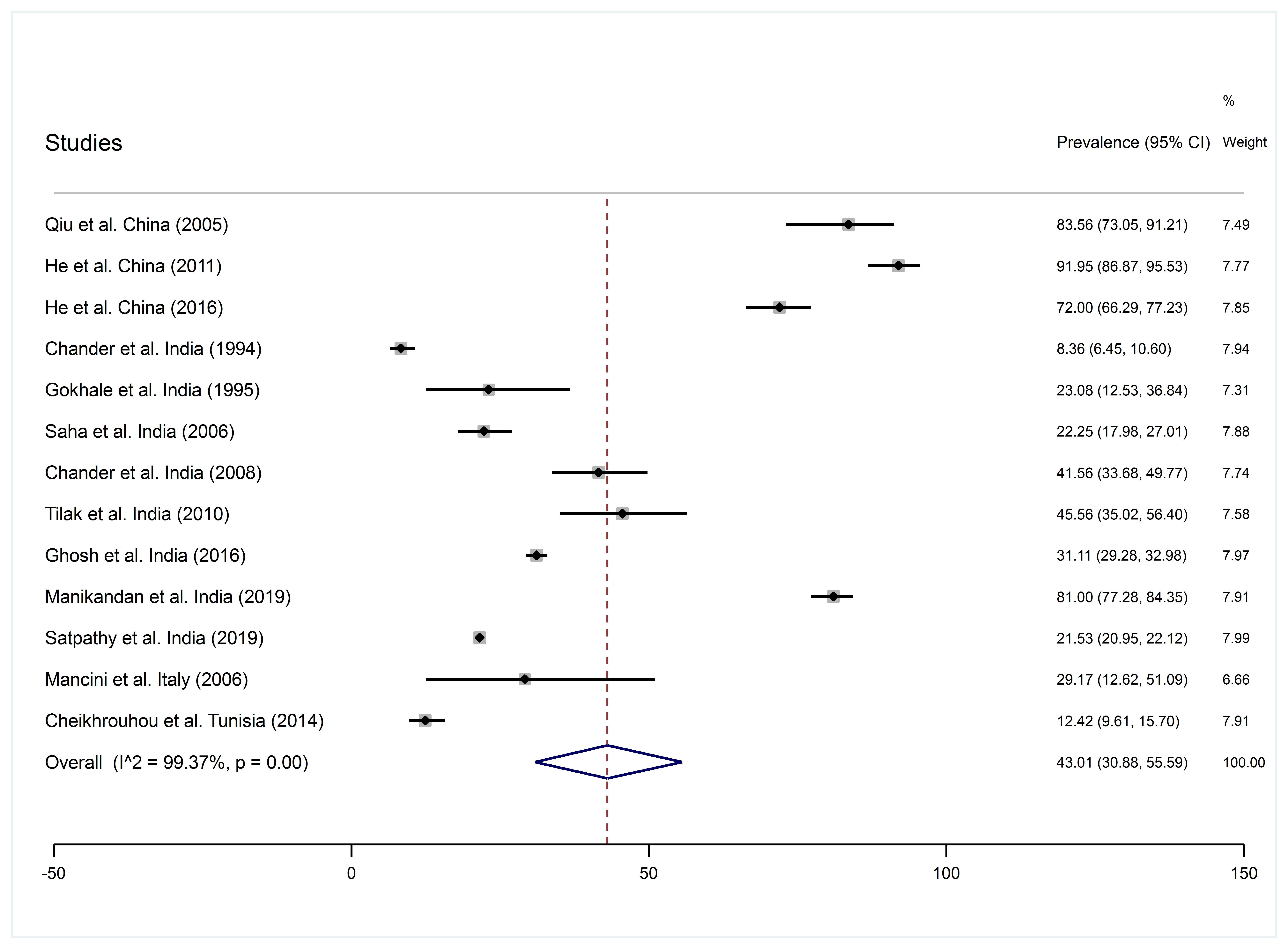

Supplement: Supplementary Figure 7 — The forest plot of the prevalence of fungal keratitis among patients with clinical suspicion of fungal keratitis based on the reported articles between January 1, 1990 and May 27, 2020. [file Image_7.jpeg]

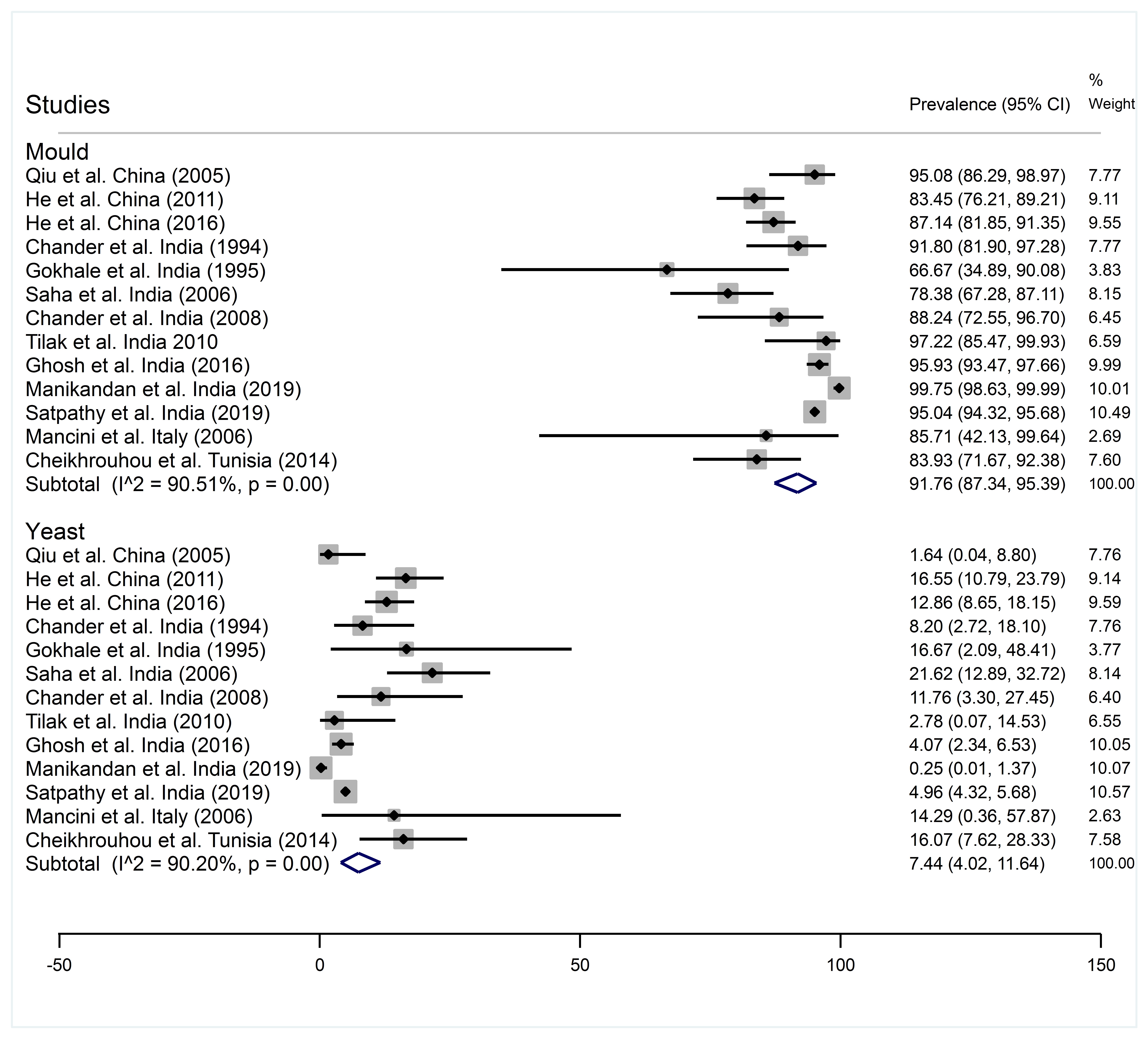

Supplement: Supplementary Figure 8 — The forest plot of the prevalence of yeast and mold keratitis among patients with clinical suspicion of fungal keratitis based on the reported articles between January 1, 1990 and May 27, 2020. [file Image_8.jpeg]

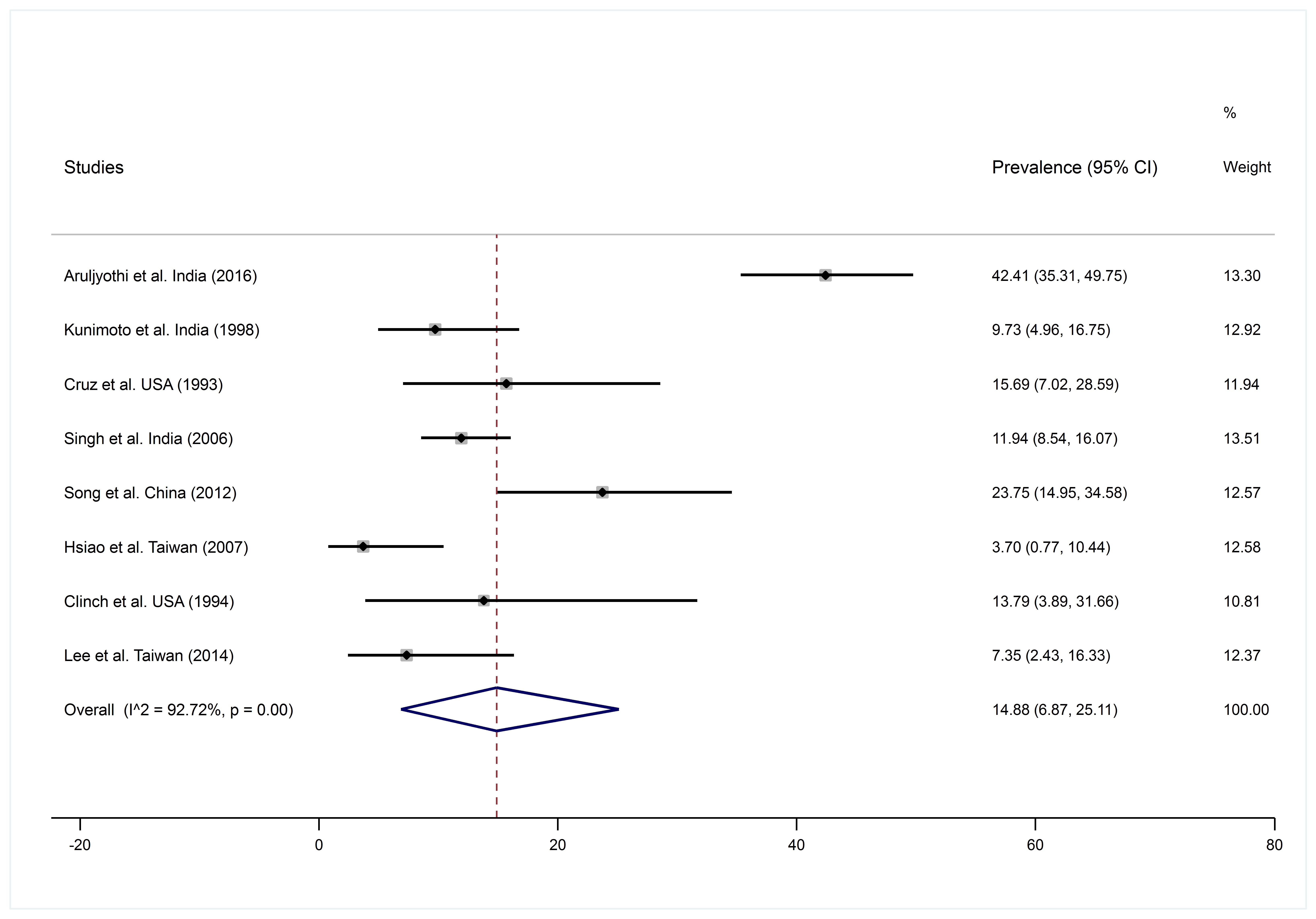

Supplement: Supplementary Figure 9 — The forest plot of the prevalence of fungal keratitis among pediatric patients based on the reported articles between January 1, 1990 and May 27, 2020. [file Image_9.jpeg]

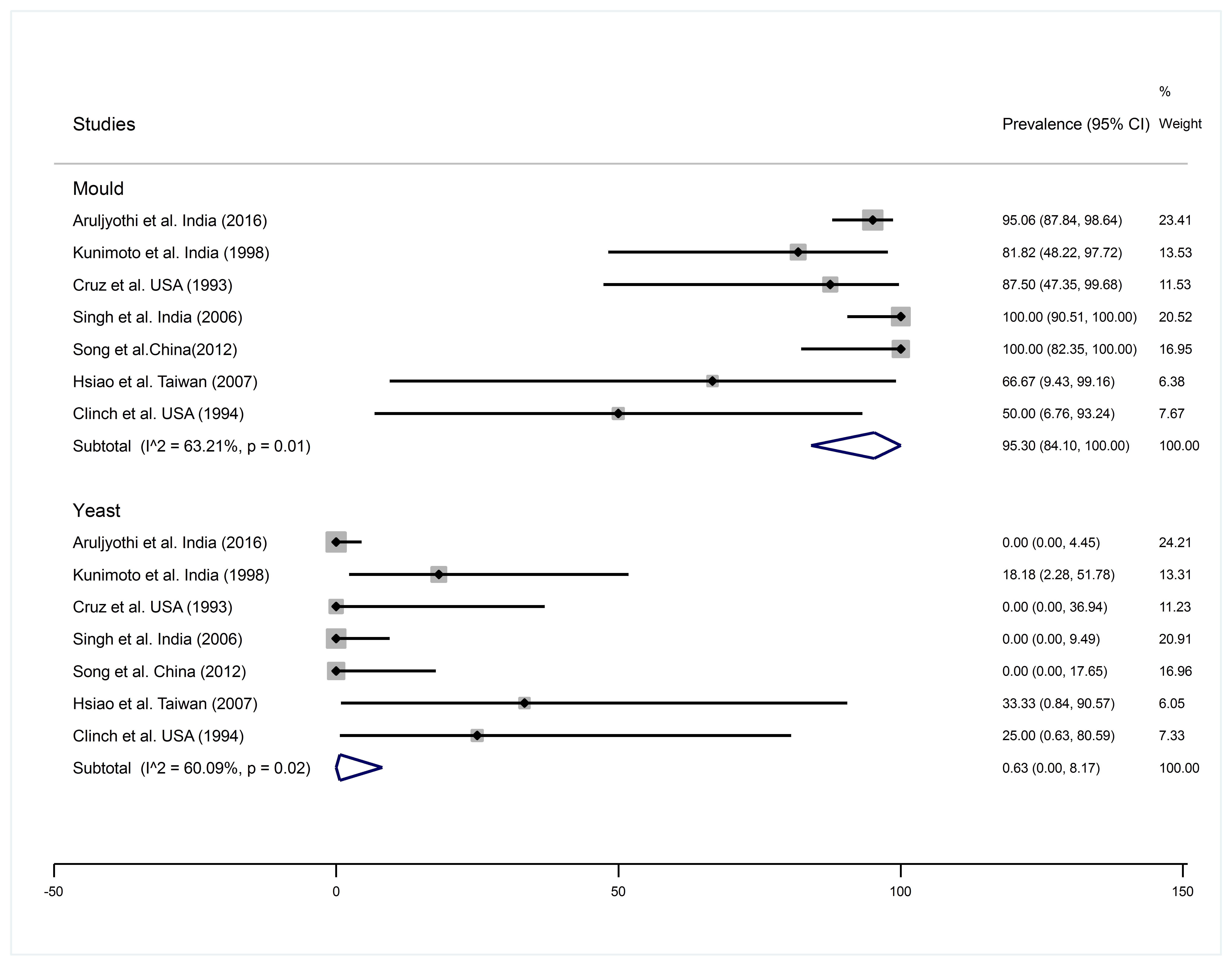

Supplement: Supplementary Figure 10 — The forest plot of the prevalence of yeast and mold keratitis among pediatric patients based on the reported articles between January 1, 1990 and May 27, 2020. [file Image_10.jpeg]

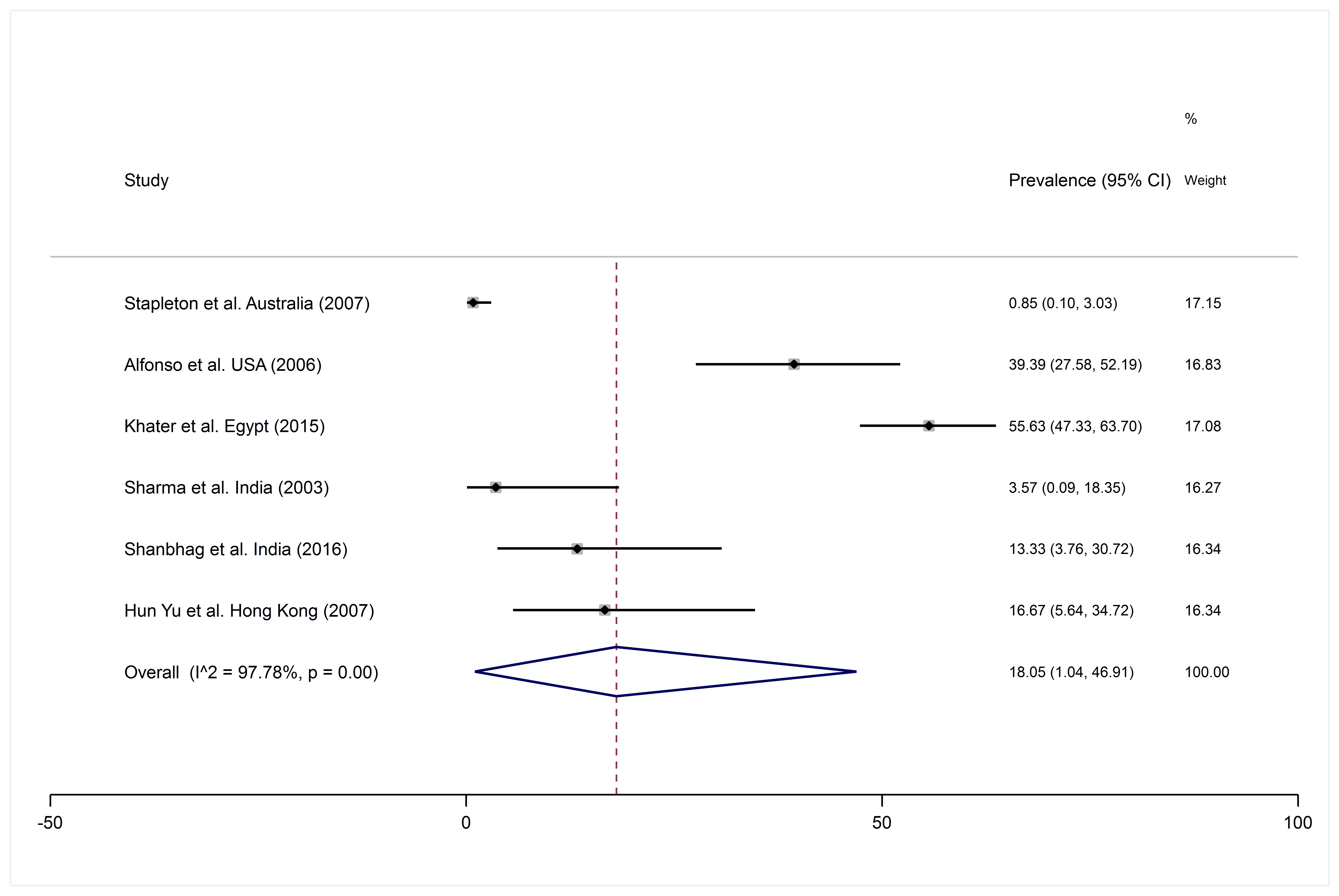

Supplement: Supplementary Figure 11 — The forest plot of the prevalence of fungal keratitis among patients with contact lens wearers based on the reported articles between January 1, 1990 and May 27, 2020. [file Image_11.jpeg]

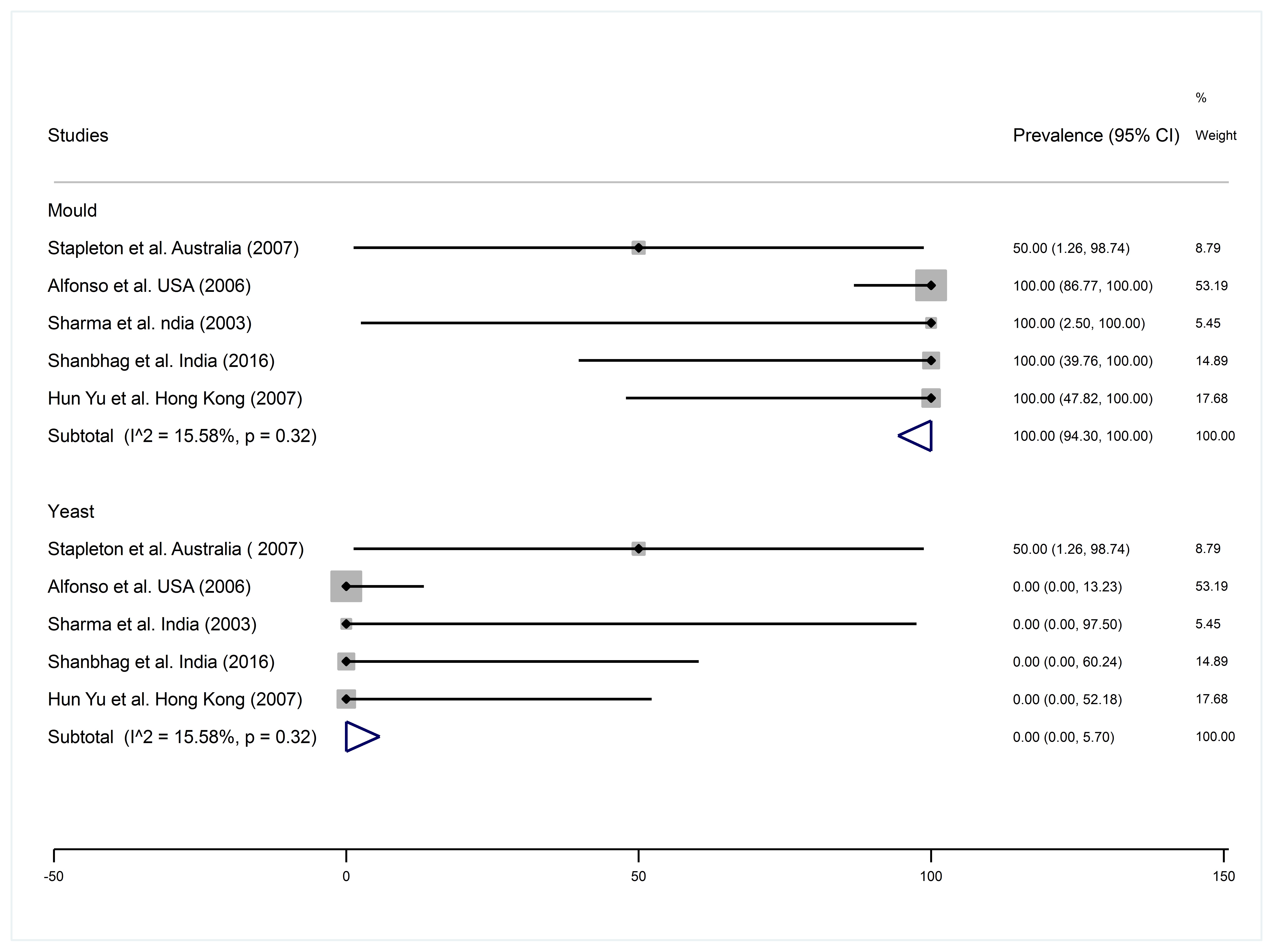

Supplement: Supplementary Figure 12 — The forest plot of the prevalence of yeast and mold keratitis among contact lens wearers based on the reported articles between January 1, 1990 and May 27, 2020. [file Image_12.jpeg]

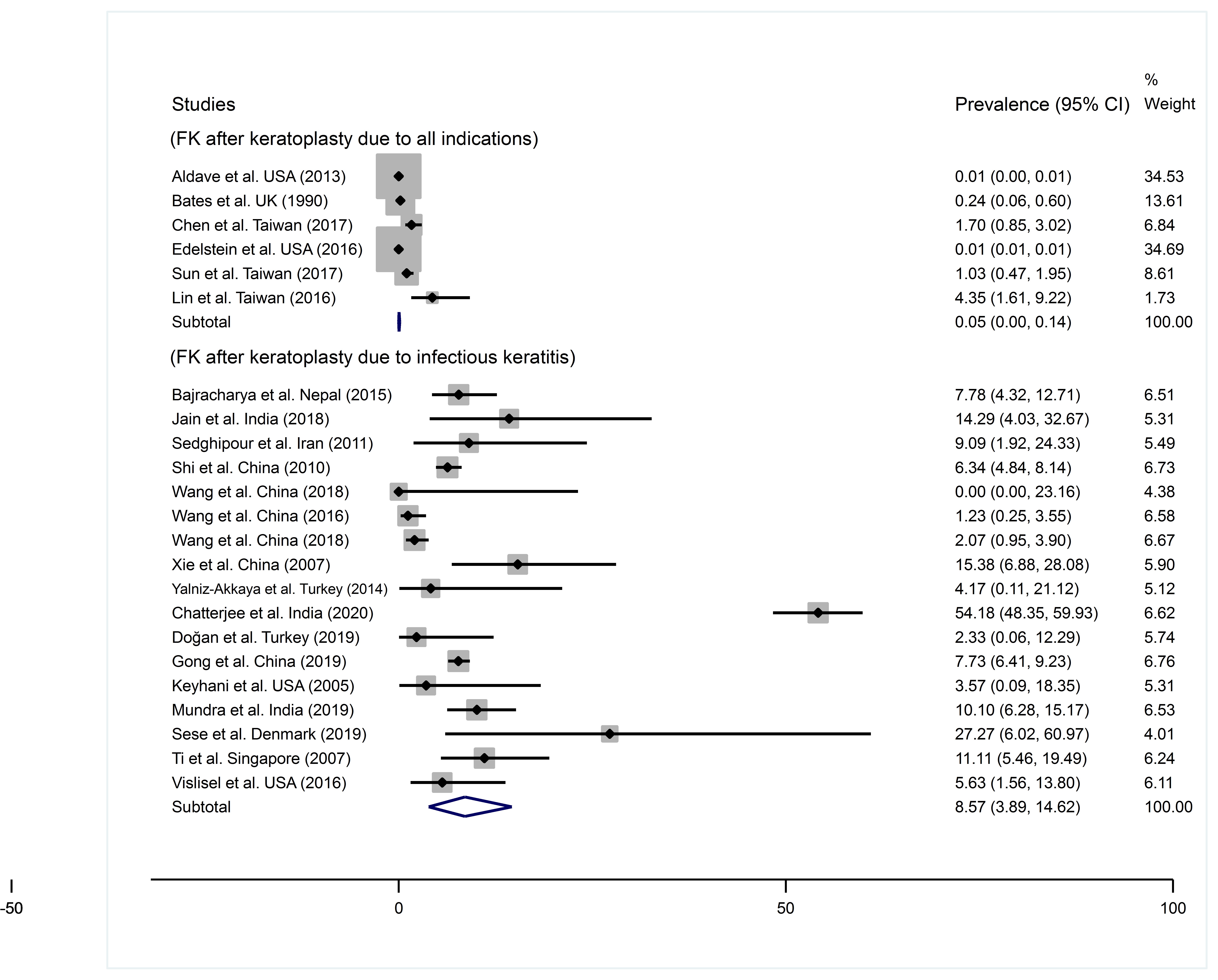

Supplement: Supplementary Figure 13 — The forest plot of the prevalence of fungal keratitis among patients undergone keratoplasty based on the reported articles between January 1, 1990 and May 27, 2020. [file Image_13.jpeg]

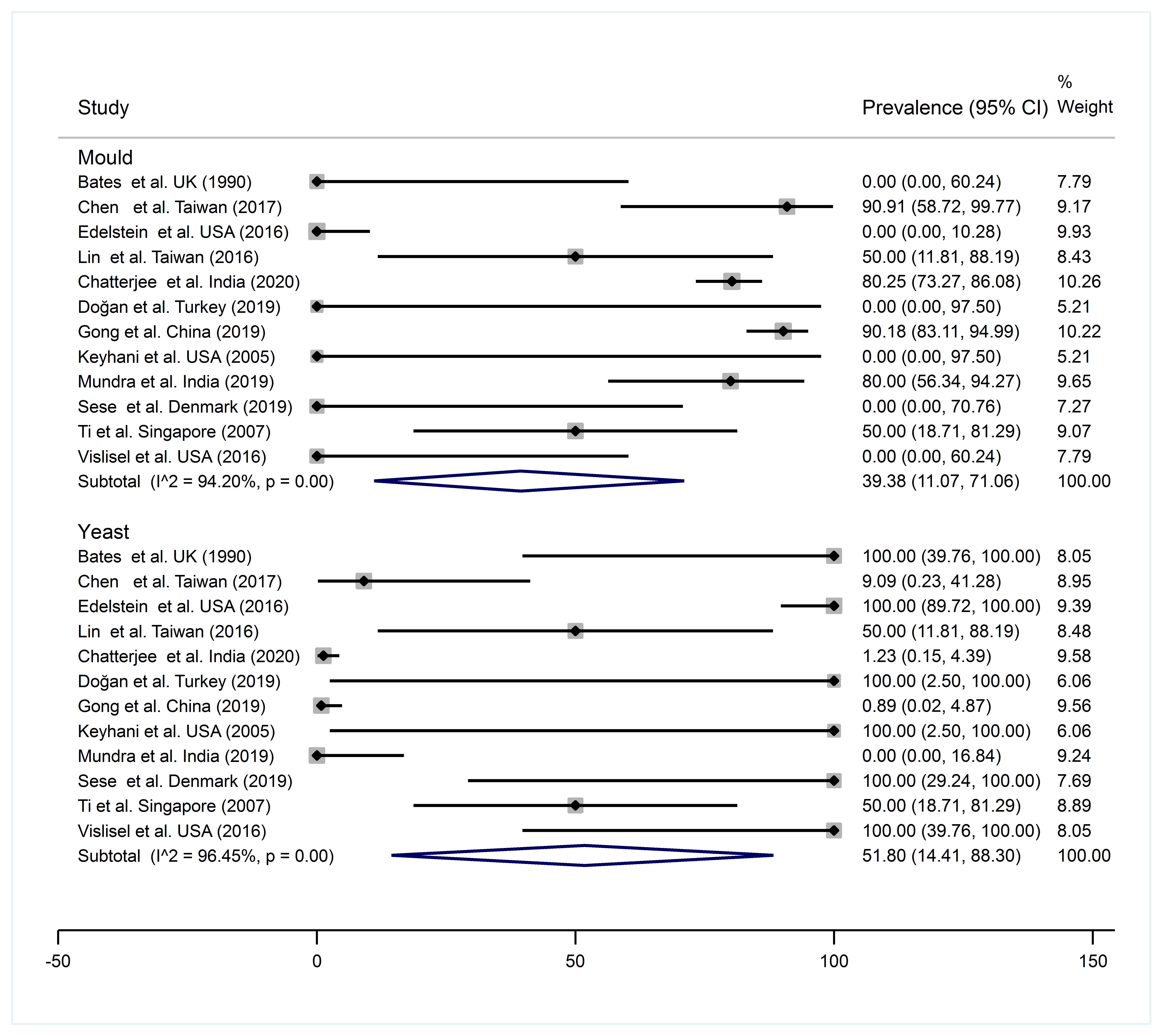

Supplement: Supplementary Figure 14 — The forest plot of the prevalence of yeast and mold keratitis among patients undergone keratoplasty based on the reported articles between January 1, 1990 and May 27, 2020. [file Image_14.jpeg]
