# Supplementary material for: Distribution, Prevalence, and Causative Agents of Fungal Keratitis: A Systematic Review and Meta-Analysis (1990 to 2020)
Source: Front Cell Infect Microbiol. 2021 Aug 26;11:698780. doi: 10.3389/fcimb.2021.698780 (PMC8428535; doi:10.3389/fcimb.2021.698780)
Supplement: Supplementary file 15 [file Table_1.docx]

| Country | N of studies | Percentage |
| --- | --- | --- |
| India | 56 | 32.94 |
| China | 16 | 9.41 |
| USA | 15 | 8.82 |
| Nepal | 13 | 7.65 |
| Taiwan | 8 | 4.71 |
| Brazil | 5 | 2.94 |
| Malaysia | 5 | 2.94 |
| Pakistan | 4 | 2.35 |
| Australia | 3 | 1.76 |
| England | 3 | 1.76 |
| Iran | 3 | 1.76 |
| Paraguay | 3 | 1.76 |
| Saudi Arabia | 3 | 1.76 |
| Thailand | 3 | 1.76 |
| Bangladesh | 2 | 1.18 |
| Egypt | 2 | 1.18 |
| Ghana | 2 | 1.18 |
| Hong Kong | 2 | 1.18 |
| Iraq | 2 | 1.18 |
| Oman | 2 | 1.18 |
| Tunisia | 2 | 1.18 |
| Turkey | 2 | 1.18 |
| Croatia | 1 | 0.59 |
| Denmark | 1 | 0.59 |
| Ethiopia | 1 | 0.59 |
| Ireland | 1 | 0.59 |
| Israel | 1 | 0.59 |
| Italy | 1 | 0.59 |
| Mexico | 1 | 0.59 |
| New Zealand | 1 | 0.59 |
| Nigeria | 1 | 0.59 |
| Sierra Leone | 1 | 0.59 |
| Singapore | 1 | 0.59 |
| Spain | 1 | 0.59 |
| Sri Lanka | 1 | 0.59 |
| Switzerland | 1 | 0.59 |
| Total | 170* | 100 |

Table S1. The distribution of eligible studies to be included in the present review based on their reporting country (1990 up to May 27, 2020)

* One article included two separate sets of data from India and Ghana. These sets were considered as different studies.
