# Supplementary material for: Distribution, Prevalence, and Causative Agents of Fungal Keratitis: A Systematic Review and Meta-Analysis (1990 to 2020)
Source: Front Cell Infect Microbiol. 2021 Aug 26;11:698780. doi: 10.3389/fcimb.2021.698780 (PMC8428535; doi:10.3389/fcimb.2021.698780)
Supplement: Supplementary file 16 [file DataSheet_1.zip › Supplementary file 2 search strategy.docx]

(((((((fungal keratitis[Title/Abstract]) OR keratitis[MeSH Terms]) OR keratomycosis[Title/Abstract]) OR mycotic keratitis[Title/Abstract]) OR fungal infection[Title/Abstract]) OR mycoses[MeSH Terms])) AND ((((eye[Title/Abstract]) OR cornea[Title/Abstract]) OR ocular[Title/Abstract]) OR corneal ulcer[Title/Abstract]) AND (("1990/01/01"[PDat] : "2020/05/27"[PDat]) AND English[lang])
